# Silencing NTPDase3 activity rehabilitates the osteogenic commitment of post-menopausal stem cell bone progenitors

**DOI:** 10.1186/s13287-023-03315-6

**Published:** 2023-04-19

**Authors:** José Bernardo Noronha-Matos, Rui Pinto-Cardoso, Catarina Bessa-Andrês, Maria Teresa Magalhães-Cardoso, Fátima Ferreirinha, Maria Adelina Costa, José Marinhas, Rolando Freitas, Rui Lemos, Adélio Vilaça, António Oliveira, Julie Pelletier, Jean Sévigny, Paulo Correia-de-Sá

**Affiliations:** 1 Laboratório de Farmacologia e Neurobiologia, Instituto de Ciências Biomédicas de Abel Salazar (ICBAS) – Universidade do Porto (UP), R. Jorge Viterbo Ferreira, 228, 4050-313 Porto, Portugal; 2 Center for Drug Discovery and Innovative Medicines (MedInUP), Porto, Portugal; 3 Departamento de Química, Instituto de Ciências Biomédicas Abel Salazar - Universidade Do Porto (ICBAS-UP), 4050-313 Porto, Portugal; 4 Serviço de Ortopedia e Traumatologia, Centro Hospitalar de Gaia - Espinho, 4434-502 Vila Nova de Gaia, Portugal; 5 Serviço de Ortopedia, Centro Hospitalar Universitário de Santo António, 4099-001 Porto, Portugal; 6 Centre de Recherche en Rhumatologie et Immunologie, University Laval, 2325, rue de l’Université Québec, Québec, G1V 0A6 Canada

**Keywords:** Mesenchymal stem cells, Post-menopausal osteogenesis, Purinergic signalling, Ectonucleotidases

## Abstract

**Background:**

Endogenously released adenine and uracil nucleotides favour the osteogenic commitment of bone marrow-derived mesenchymal stromal cells (BM-MSCs) through the activation of ATP-sensitive P2X7 and UDP-sensitive P2Y_6_ receptors. Yet, these nucleotides have their osteogenic potential compromised in post-menopausal (Pm) women due to overexpression of nucleotide metabolizing enzymes, namely NTPDase3. This prompted us to investigate whether NTPDase3 gene silencing or inhibition of its enzymatic activity could rehabilitate the osteogenic potential of Pm BM-MSCs.

**Methods:**

MSCs were harvested from the bone marrow of Pm women (69 ± 2 years old) and younger female controls (22 ± 4 years old). The cells were allowed to grow for 35 days in an osteogenic-inducing medium in either the absence or the presence of NTPDase3 inhibitors (PSB 06126 and hN3-B3_s_ antibody); pre-treatment with a lentiviral short hairpin RNA (Lenti-shRNA) was used to silence the NTPDase3 gene expression. Immunofluorescence confocal microscopy was used to monitor protein cell densities. The osteogenic commitment of BM-MSCs was assessed by increases in the alkaline phosphatase (ALP) activity. The amount of the osteogenic transcription factor Osterix and the alizarin red-stained bone nodule formation. ATP was measured with the luciferin-luciferase bioluminescence assay. The kinetics of the extracellular ATP (100 µM) and UDP (100 µM) catabolism was assessed by HPLC

**Results:**

The extracellular catabolism of ATP and UDP was faster in BM-MSCs from Pm women compared to younger females. The immunoreactivity against NTPDase3 increased 5.6-fold in BM-MSCs from Pm women vs. younger females. Selective inhibition or transient NTPDase3 gene silencing increased the extracellular accumulation of adenine and uracil nucleotides in cultured Pm BM-MSCs. Downregulation of NTPDase3 expression or activity rehabilitated the osteogenic commitment of Pm BM-MSCs measured as increases in ALP activity, Osterix protein cellular content and bone nodule formation; blockage of P2X7 and P2Y_6_ purinoceptors prevented this effect.

**Conclusions:**

Data suggest that NTPDase3 overexpression in BM-MSCs may be a clinical surrogate of the osteogenic differentiation impairment in Pm women. Thus, besides P2X7 and P2Y_6_ receptors activation, targeting NTPDase3 may represent a novel therapeutic strategy to increase bone mass and reduce the osteoporotic risk of fractures in Pm women.

**Supplementary Information:**

The online version contains supplementary material available at 10.1186/s13287-023-03315-6.

## Introduction

Bone marrow-derived mesenchymal stem cells (BM-MSCs) exhibit extensive proliferative ability in an uncommitted state, while retaining the great potential to differentiate into various cell lineages (e.g. osteoblasts, adipocytes and chondrocytes) [1–4]. BM-MSCs retain their proliferative potential throughout life, yet their ability to differentiate into bone-forming osteoblasts is compromised in post-menopausal (Pm) women [5, 6], leading to unbalanced bone resorption by osteoclasts.

Extracellular adenine and uracil nucleotides play important roles in the regulation of bone formation [7]. Nucleotides are constitutively released from BM-MSCs and differentiated osteoblasts when submitted to mechanical load, low-intensity pulsed ultrasound, or in the presence of chemical stimulants, like 1,25(OH)_2_ vitamin D3 and bisphosphonates [8–13], which may explain their relevance in bone remodelling. The extracellular nucleotide levels increase dramatically during bone injury, which is paramount to bone remodelling and fracture healing [8, 14, 15]. Epidemiological, pharmacological and molecular data suggest that the loss of function of the ATP-sensitive P2X7 receptor reduces bone mineral density and increases the risk of fractures in Pm women [5, 16, 17]. Besides ATP, also uracil nucleotides are important regulators of osteogenic differentiation of Pm BM-MSCs through the activation of UDP-sensitive P2Y_6_ receptors [6]. Conflicting results, however, exist implicating the influence of pyrimidine-sensitive P2Y_2_ and P2Y_4_ receptors in bone human formation [18–21].

The effects of endogenously released adenine and uracil nucleotides by BM-MSCs are normally balanced by the activity of several plasma membrane-bound NTPDases, which catalyse their extracellular breakdown and, thereby, determine whether osteoblast progenitors are driven into proliferation or differentiation. Previous findings from our group showed that BM-MSCs from Pm women exhibit deficits in the activation of P2X7 and P2Y_6_ receptors [5, 6], but this was not observed in cells obtained from younger females [5]. Membrane compartmentalization of ectoenzymes, nucleotide releasing sites and purinoceptors [22] emphasizes the value of gathering information concerning the kinetics of the extracellular catabolism of adenine and uracil nucleotides by NTPDase subtypes, along with the subsequent delivery of their metabolites to specific P2 purinoceptors, to predict their relevance in bone remodelling.

Our working hypothesis is that age- (and/or pathophysiological)-related changes in the expression and activity of substrate-specific NTPDases might have a significant impact on Pm bone formation. Increasing evidence demonstrates that targeting the activity of NTPDases may be therapeutically valuable in blood clotting, vascular inflammation, immune reactions, and certain types of cancer [23]. Here, we tested whether changes in the expression and/or activity of NTPDase subtypes contribute to the osteogenic differentiation deficits of BM-MSCs obtained from Pm women compared to those isolated from younger females. Data show that NTPDase3 overexpression emerges as a distinctive surrogate of osteogenic impairment of BM-MSCs in Pm women compared to other NTPDase subtypes. Thus, normalization of NTPDase3 expression levels and/or inhibition of its activity may represent novel therapeutic approaches to promote osteogenesis and increase bone formation in Pm women.

## Materials and methods

### Reagents, lenti-shRNAs and antibodies

ATP, quinacrine mustard dihydrochloride, 3,4-dihydroxy-9,10-dioxo-2-anthracenesulfonic acid sodium salt (Alizarin red S), 3-[4,5-dimethylthiazol-2-yl]-2,5-diphenyl tetrasodium bromide (MTT), 4-nitrophenyl phosphate and UDP were obtained from Sigma-Aldrich (St. Louis, MO, USA). 3-[[5-(2,3-dichlorophenyl)-1H-tetrazol-1-yl]methyl]pyridine hydrochloride (A438079), N,N′′-1,4-butanediylbis[N′-(3-isothiocyanate phenyl) thiourea (MRS 2578) and 1-amino-4-(1-naphthyl)aminoanthraquinone-2-sulfonic acid sodium salt (PSB 06126) were obtained from Tocris Cookson Inc. (Bristol, UK). All primary anti-human and secondary conjugated antibodies used in this study have been previously validated [6, 24–26]. Anti-β-actin antibody (rabbit), anti-GAPDH (glyceraldehyde-3-phosphate dehydrogenase; mouse), anti-Osterix (rabbit), horseradish-peroxidase-conjugated secondary antibodies (anti-rabbit and anti-mouse) were obtained from Abcam (Cambridge, UK); anti-IgG2a (mouse) and anti-IgG2b (mouse) were purchased from BioLegend (San Diego, CA, USA); anti-human ecto-NTPDases antibodies, CD73 antibody and pre-immune rabbit and guinea pig serums were developed by JP and JS at the Centre de Recherche en Rhumatologie et Immunologie, University of Laval, Québec, QC, Canada (see http://ectonucleotidases-ab.com for further details); primary antibodies included: h5′NT-2_L_I_5_ (rabbit), hN1-1_C_I_5_ (guinea pig), hN2-kw3I4 (rabbit), hN3-1_C_I_4_ (guinea pig), hN3-B3_S_ (mouse) and hN8-C5_S_ (mouse). Alexa Fluor 488-labelled anti-rabbit, Alexa Fluor 568-labelled anti-mouse and Alexa Fluor 649-labelled anti-guinea pig were supplied by Molecular Probes (Invitrogen, Carlsbad, CA, USA). TRITC 568-labelled anti-guinea pig was purchased from Jackson ImmunoResearch (Newmarket, Suffolk, UK). pGFP-C-shLenti Vectors were supplied from ORIGENE (Rockville, MD, USA).

### Cell culture conditions and phenotypic characterization of the cells

Human cancellous bone marrow samples were obtained (i) from the neck of the femur of thirty consecutive female patients (69 ± 2 years old, Pm group) undergoing total hip arthroplasty as a result of non-inflammatory degenerative osteoarthrosis, and (ii) from the iliac crest or sacrum of eight younger female patients (22 ± 4 years old, control group) requiring bone engraftment for spinal fusion to correct scoliosis or fracture osteosynthesis. For comparison purposes, cancellous bone marrow samples were also obtained from two men, one matching the age of the younger female group and the other pairing with the Pm group. Handling of bone marrow samples and culture of adherent cells was performed until near confluence for 10–15 days, as previously described [5, 6]. To avoid the influence of in vitro cells senescence and phenotypic modifications, we used only the first subcultures made with 2.5 × 10^4^ cells/mL density, which were maintained for 35 days in standard culture medium (α-minimal essential medium, α-MEM) plus 10% foetal bovine serum, 100 U/mL penicillin, 100 μg/mL streptomycin and 2.5 μg/mL amphotericin B supplemented with 50 μg/mL ascorbic acid, 10 mM β-glycerophosphate and 10 nM dexamethasone to promote osteogenic differentiation. Bone marrow cell cultures were carried out for 35 days in either the absence (control) or the presence of the test drugs, which were added to the culture medium from day 1 onwards [3, 5, 6]. Drugs were renewed in the culture at each medium change, i.e. twice a week. No pooled samples obtained from different individuals were used for any of the protocols described in this study.

Phenotypic characterization of the cells (first subculture) was performed by flow cytometry [6]. These cells exhibited positive immunoreactivity against CD105 (SH2), CD29 (integrin ß1) and CD117 (tyrosine-protein kinase Kit), which have been identified as surface markers of BM-MSCs [1, 2, 4, 27–30]. Conversely, the cells were negative for haematopoietic surface markers, like CD14 and CD45, which have been extensively used as a good argument to distinguish bone marrow haematopoietic cells from MSCs [4, 27]. Thus, first passage plastic-adherent human bone marrow cells obtained under the present experimental conditions are highly enriched in multipotent MSCs. See e.g. Ref [6].

### NTPDase3 gene silencing using a lentivirus-coupled shRNA

For NTPDase3 gene silencing, the first sub-cultures were treated with pGFP-C-shLenti Vectors (ORIGENE – code TL313202V) against NTPDase3; 4 specific sequences were used, namely: TL313202VA (CTCTACACACACAGCTTCCAGTGCTATGG), TL313202VB (ATTTCCTGGAGAAGAACCTGTGGCACATG), TL313202VC (CGCTCTTACTGCTTCTCAGCCAACTACAT) and TL313202VD (GCAGGATTCTACTACACAGCCAGTGCTTT). A mismatch sequence was used as a negative control (scramble, TR30021V). Cells were incubated with the lenti-shRNAs for 24 h; to promote more extensive cell transduction, half the volume of the cells medium was used during the incubation period for all 4 specific and scrambled sequences, as well as for control cultures (no treatment) [31]. During validation experiments, we used increasing multiplicities of infection (MOI), namely 1, 3 and 10 (see Additional file 1: Fig. S2); we found that sequence TL313202VD (MOI 3) was the most promising NTPDase3 gene silencing condition, given that (i) it was able to significantly decrease NTPDase3 protein levels; (ii) the scramble sequence was unable to change NTPDase3 protein levels (see Additional file 1: Fig. S2); (iii) no difference was found in the number of viable cells between scramble and shRNA treated cells (data not shown). After a 24 h incubation period, cell cultures were washed three times with phosphate-buffered saline (PBS, 1x) and then maintained for 35 days in supplemented cell medium as indicated above [31].

### Viability/proliferation and osteogenic differentiation of BM-MSCs

Cell viability/proliferation was evaluated by the MTT assay [3, 5, 6]. Data from the MTT assay correlates positively with the results measuring cell proliferation from total DNA quantification per culture well [6]. Osteogenic differentiation of BM-MSCs was inferred as increases in alkaline phosphatase (ALP) activity and the expression of the osteogenic transcription factor Osterix. ALP activity was determined in cell lysates by colorimetric determination of *p*-nitrophenyl phosphate (PNP) hydrolysis, as previously described [3, 5, 6, 32]; obtained values were expressed in nmol of PNP per min normalized by the MTT value (nmol min^−1^ MTT^−1^) [5] and presented as percentage of controls (100%).

Total amounts of Osterix protein were determined by Western blot analysis at culture day 21 [5]. Equal protein amounts (25 µg) were loaded into sodium dodecyl sulphate–polyacrylamide gel electrophoresis (SDS-PAGE) (10%) gels and transferred onto a polyvinylidene fluoride membrane using a Mini-Protean Tetra Cell coupled to a Mini-Trans-Blot module (Bio-Rad, Hercules, CA, USA). Blocked membranes were incubated with the anti-human primary antibody (1:200) anti-Osterix (rabbit); anti-β-Actin (1:1000, rabbit) or anti-GAPDH (1:200, mouse) were used for normalization purposes (i.e. Osterix/β-Actin and Osterix/GAPDH). The peroxidase detection system (1.25 mM luminol, 0.2 mM coumaric acid, 0.1 M Tris pH 8.5, 0.032% hydrogen peroxide) was used for visualization of the immunoreactivity, using the horseradish-peroxidase-conjugated secondary antibodies (1:70000). Gels were analysed using a gel blot imaging system (ChemiDoc MP, Bio-Rad, Hercules, CA, USA).

At culture day 35, calcium deposition in mineralized nodules was revealed by the Alizarin Red staining and photographed using an optic microscope (Olympus CKX41, Tokyo, Japan) equipped with a digital camera (Olympus SC30, Tokyo, Japan), running an image acquisition software (Olympus Analysis GetIT 5.1, Tokyo, Japan) [5]; calibrated images were exported to Image J 1.37c software (NIH, Bethesda, MD, USA) for quantification of total bone-nodule areas. Results are expressed as percentages relative to controls (100%) [5, 33].

### Immunofluorescence staining

Human BM-MSCs were allowed to grow in chamber slides for 7 or 21 days. Paraformaldehyde fixed cells were incubated in the dark for 2 h with the following primary antibodies: ecto-5′-nucleotidase 1:1000 (h5′NT-2_L_I_5_, rabbit), NTPDase1 1:1000 (hN1-1_C_I_5_, guinea pig), NTPDase2 1:200 (hN2-Kw3I4, rabbit), NTPDase3 1:200 (hN3-B3_S_, mouse; hN3-1_C_I_4_, guinea pig) and NTPDase8 1:1000 (hN8-C5_S_, mouse). Alexa Fluor 488 1:1500 (anti-rabbit), Alexa Fluor 568 1:1500 (anti-mouse), Alexa-Fluor 649 1:1500 (anti-guinea pig) and TRITC 1:150 (anti-guinea pig) were applied as secondary antibodies for 1 h in the dark. Glass slides were mounted with VectaShield mounting medium and stored at 4 °C. Observations were made using a laser-scanning spectral confocal microscope (Olympus FV1000, Tokyo, Japan) built on an IX81SF-3 inverted motorized microscope with four laser lines controlled by an AOTF laser combiner. Both multi-argon laser and diode laser 405 lines, filtered by barrier filters Ion Coating for OLYMPUS UIS-2 optics, through a UPLSAPO40xOl / NA 0.5–1.0 WD 0.12 mm objective lens (Olympus, Tokyo, Japan), were used to acquire images unless otherwise stated. The Fluoview FV1000 Advanced Software (4.0.3.4 version; Olympus, Tokyo, Japan) was used to analyse data and to control image acquisition parameters, which were set to one-way XY repeat scanning mode at 12.5 s/pixel speed with the pinhole set to 250 µm at an image resolution of 640 × 640 pixel (317.583 × 317.583 µm given that 1 pixel = 0.497 µm). Acquired micrographs were stored in the Olympus Multi TIFF format (Tokyo, Japan) [3, 5, 6, 34]. For comparison purposes, confocal microscope settings and image acquisition parameters were kept unaltered throughout parallel documentation procedures. Negative controls were performed in the absence of the primary antibodies; replacement of primary antibodies by pre-immune sera (rabbit, guinea-pig) or by corresponding IgG2a and IgG2b antibodies, was also made in certain conditions (see Additional file 2: Fig. S3). Five microscopic fields (area of approx. 93,000 µm^2^ each) were photographed per well. Standardization of XY image coordinates was as follows: the first image was taken at the centre of the well (*X* = 0; *Y* = 0) and the next four images were obtained sequentially from each corner of a hypothetical square enclosed in a circumference of 0.275 cm radius. The obtained five independent images were exported to Image J 1.37c software (NIH, Bethesda, MD, USA) for quantification analysis. Regions of interest (ROIs) outlining complete individual cells were done manually and the average intensity of the pixels inside each cell was calculated per micrograph. The background fluorescence estimated from outlined ROIs drawn without transecting any cell was subtracted from all monitored ROIs. The computed analysis of the five individual images was expressed as the average fluorescence intensity (arbitrary units, a.u.) for each experimental condition. Shown in the figures is typical immunofluorescence images for each experimental condition, taken from a single representative microscopic field without juxtaposition. When necessary, software adjustments were applied to the entire image.

### Kinetic experiments and HPLC analysis

The kinetics of the extracellular catabolism of ATP and UDP in human primary BM-MSC cultures was evaluated, at 37ºC, on days 7 and 21 [3, 6]. After a 30-min equilibration period, the cells were incubated with gassed (95% O_2_ plus 5% CO_2_) Tyrode’s solution (137 mM NaCl, 2.7 mM KCl, 1.8 mM CaCl_2_, 1 mM MgCl_2_, 0.4 mM NaH_2_PO_4_, 11.9 mM NaHCO_3_, and 11.2 mM glucose, pH 7.4) supplemented with 100 µM ATP or UDP (zero time). When testing the effect of NTPDase inhibitors, PSB 06126 (3 µM) or hN3-B3_S_ (0.5 µg/ml), these drugs were added to the incubation solution 15 min before application of ATP and UDP. Samples (75 μl) were collected from each well at different times up to 30 min for HPLC analysis (LaChrom Elite, Merck, Frankfurt, Germany) of the variation of substrate disappearance and product formation; 20-µl injection volumes were used for the analysis. The concentrations of the substrates and their respective metabolites were plotted as a function of time (progress curves, see e.g. Figure 4). The following parameters were analysed for each progress curve: half-life time (*t*_*1/2*_) of the initial substrate, time of appearance of the different concentrations of the products, the concentration of the substrate or any product remaining at the end of the experiment, and the slope variation of ATP and UDP catabolism (rate of disappearance) obtained by linear regression between 0 and 15 min (linear region of the curve; Fig. 4). The enzymatic activity was normalized to the number of viable cells given by the MTT assay (Fig. 2; cf [6].). The spontaneous degradation of ATP and UDP, at 37ºC, was negligible over a 30-min time in the absence of the cells. At the end of the experiments, the remaining incubation medium was collected and used to quantify the lactate dehydrogenase (LDH, EC 1.1.1.27) activity [35]. The negligible activity of LDH in the samples collected at the end of the experiments is an indication of the integrity of the cells during the experimental procedure.

### Quinacrine-stained intracellular ATP deposits and extracellular ATP bioluminescence

We used quinacrine fluorescence staining (ex: 476 nm / em: 500–540 nm) to visualize ATP intracellular stores. To this end, cells were allowed to grow for 7 days in an osteogenic-inducible medium. After removing the incubation medium, the cells were washed three times with phosphate-buffered saline (PBS, 1x) and subsequently incubated for 1 h with quinacrine (30 µM), at 37 °C [25]. Images were acquired using an epifluorescence microscope equipped with an XBO 75W Xenon arc lamp (Achroplan; Zeiss, Oberkochen, Germany). The light path included ET460/30 × excitation / ET520/40 m emission filters (Chroma Technology Corp, Bellows Falls, VT, USA) and a LUMPLFLN40XW/0.80NA/3.3WD water dipping objective lens (Olympus, Tokyo, Japan). A high-resolution cooled CCD camera (CoolSnap HQ, Roper Scientific Photometrics, Tucson, AZ, USA) connected to a computer running a digital image acquisition software (MetaFluor 6.3; Molecular Devices Inc., Sunnyvale, CA, USA) was used to record images in the TIFF format. The CCD exposure time was set to 100 ms, binning was adjusted to 2 and gain to 1.

Extracellular ATP was quantified using the luciferin-luciferase ATP bioluminescence assay kit HS II (Roche Applied Science, Indianapolis, Indiana, USA) in a multi-detection microplate reader (Synergy HT, BioTek Instruments, Vermont, USA), as described elsewhere [25]. Briefly, cells were seeded onto 96-well microplates, at a density of 2.5 × 10^4^ cells/mL, for 7 days (2–4 replicas were performed per individual experiment). At the beginning of the experiment, cells were washed twice with Tyrode’s solution and allowed to equilibrate for 30 min, at 37 °C. After equilibrium, cells were washed again and incubated with Tyrode’s solution in the absence or the presence of test drugs; 30 min later, the incubation fluid was removed and snap-frozen in liquid nitrogen. Before adding the luciferin-luciferase mixture, collected samples were defrosted until 25ºC according to the manufacturer's instructions. Sample bioluminescence was compared to external high-purity ATP standards; these were prepared daily within the same concentration range; all samples were run in duplicate. The remaining incubation medium was used to quantify the LDH (EC﻿ 1.1.1.27) activity [35], to evaluate cell integrity during the experimental period (see e.g. Ref. [25]). The LDH activity was negligible (< 0.1 mU/mL) in all measured samples indicating the integrity of the cells during the experimental period.

### Presentation of data and statistical analysis

Data are expressed as mean ± S.E.M. from an *n* number of individuals. Due to restricted access to similar human samples and a limited pool of initial cell density, we were unable to perform all the indicated assays in all collected human samples. Therefore, immunoblot data are expressed as individual bars to show inter-individual variability and the lack of influence of the protein used for normalization purposes. Normality tests included D'Agostino & Pearson and Shapiro–Wilk, depending on sample size. According to normality test results, statistical analyses included parametric (one-way ANOVA with Sidak’s test for multiple comparisons or the two-tailed unpaired *t*-test for double comparisons) or nonparametric tests (the Wilcoxon signed rank test for comparing the median of a set of values against a hypothetical median), besides, with a confidence level of 0.05 (95% confidence interval). Values of *P* < 0.05 were considered to represent significant differences.

## Results

### *Age-related differences in the expression of NTPDases and ecto-5*′*-nucleotidase/CD73 in human BM-MSCs undergoing osteogenic differentiation*

Using the MTT assay and DNA quantification methods, we showed that cultured human BM-MSCs proliferate in osteogenic-inducing media until near confluence is reached at culture day 21–28. While BM-MSCs retain their proliferative potential throughout life, the cells originating from Pm women have decreased ability to differentiate into bone-forming osteoblasts compared to those from younger females [6]. Although usage of different collection bone sites among the two women groups may be a limitation, the observed changes can hardly be explained solely by this bioethical constraint because care was taken to strictly use cancellous bone samples outside the subchondral bone region that would otherwise be influenced by the non-inflammatory degenerative joint disease process affecting Pm women samples, but not those from younger females. Here, we questioned whether the lack of tonic activity of P2X7 and P2Y_6_ receptors and, thereby, the purinergic-induced osteogenic commitment of Pm BM-MSC could be due to changes in the expression and activity of extracellular nucleotide metabolizing enzymes, namely NTPDases [6], compared to cells of younger females.

**Fig. 1:**
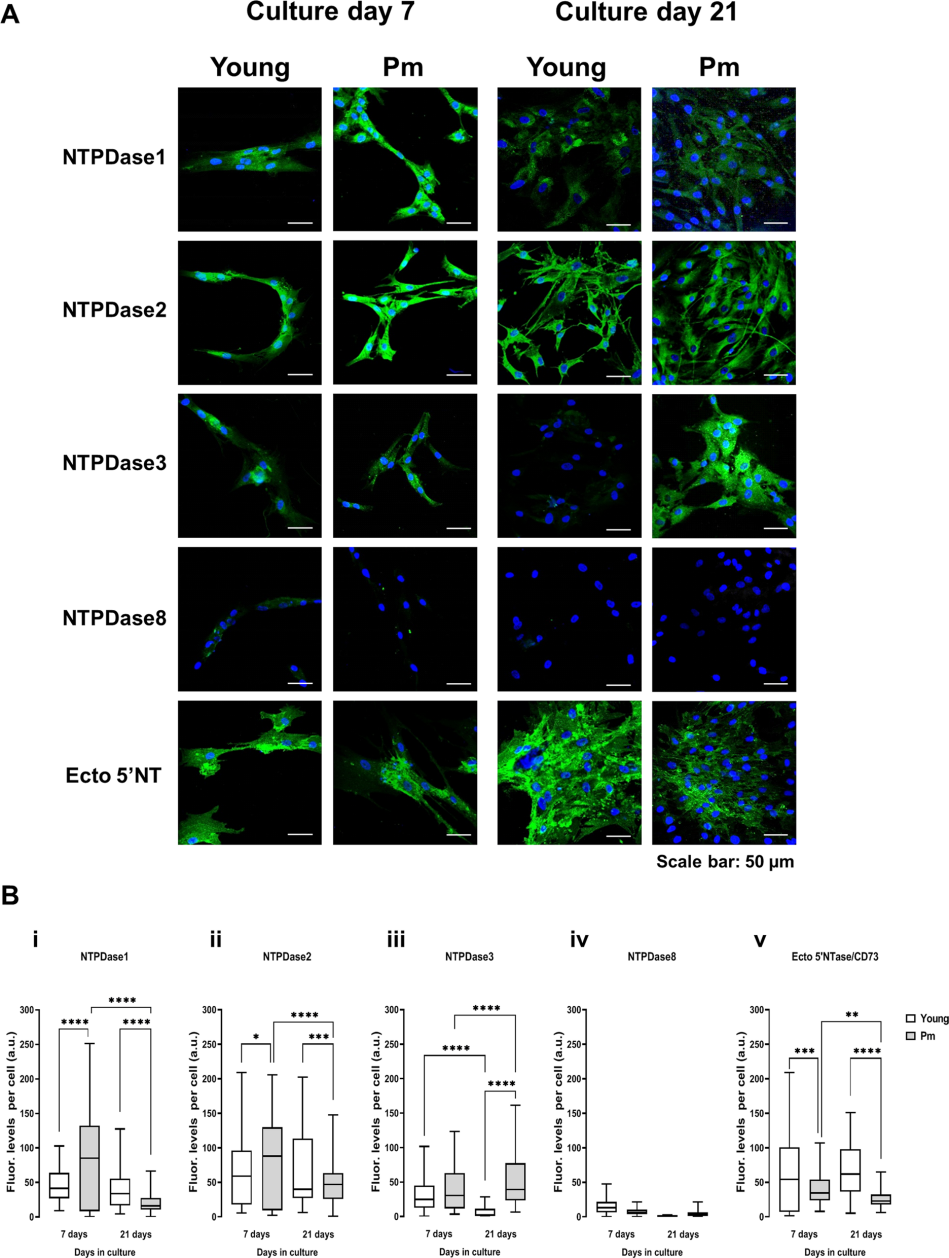
The pattern of NTPDases expression in human BM-MSCs. Panel A presents representative images of the immunocytochemical detection of NTPDase1, −2, −3, −8 and ecto-5′-nucleotidase (Ecto 5'NTase/CD73) in cultured BM-MSCs (first subculture) obtained from a young female and a Pm woman. Shown is the time-related immunoreactivity (green) detected by confocal microscopy in BM-MSCs allowed to grow for 7 and 21 days in an osteogenic-inducing medium. Blue dots represent nuclei stained with DAPI. Experiments were performed in parallel keeping unaltered the settings of the confocal microscope throughout the documentation procedure (see Materials and Methods). The scale bar is 50 µm. In panel B, shown are immunofluorescence intensity graphs computed from confocal microscopy images acquired as in panel A. Ordinates represent fluorescence intensity per cell (arbitrary units, a.u.) of enzymes immunoreactivity as a function of the number of days in culture (day 7 and 21). Regions of interest (ROIs, corresponding to individual BM-MSCs) were manually outlined and the average intensity of the pixels inside this area was calculated. Background fluorescence was estimated from outlined regions (with no cells) and subtracted from all monitored ROIs. A total of 58–234 cells were analysed from three young females (21 ± 7 years old) and six Pm women (63 ± 4 years old). *P < 0.05, **P < 0.01, ***P < 0.001 and ****P < 0.0001 (one-way ANOVA with Sidák’s multiple comparison test, single pooled variance) represent significant differences

Pm BM-MSCs show immunoreactivity against NTPDase1, -2, and -3 (Fig. 1A¸ see also [6]); while NTPDase1 and NTPDase2 enzymes were more expressed at early culture stages (day 7), NTPDase3 becomes the most represented enzyme at culture day 21 (Fig. 1A and B). This scenario differs from that observed in osteogenic differentiating cells obtained from younger females, in which NTPDase1 and -2 protein amounts remain high throughout the culture period (7 = 21 days); the Pm/young females ratio ranged from 0.53 to 0.72 concerning NTPDase1 and -2 protein amounts at culture day 21, respectively (Fig. 1A and B). The most striking difference relates to the expression of NTPDase3. While NTPDase3 protein amounts progressively increased with time in cells from Pm women (7 < 21 days), this enzyme almost disappeared from differentiated BM-MSCs of younger females (7 > 21 days); BM-MSCs from Pm women display 5.6-fold higher NTPDase3 amounts compared to the cells from younger females at culture day 21 (Fig. 1A and Biii). We confirmed this phenotypic divergence using two distinct NTPDase3 antibodies, including the previously available hN3-1_C_I_4_ polyclonal antibody [6] and a newer highly-specific hN3-B3_S_ monoclonal antibody (Fig. 1A) which is also instrumental as an enzyme inhibitor [26]. The absence of NTPDase3 immunoreactivity in BM-MSCs from age-matched males, while keeping a similar density pattern regarding other ectonucleotidases (see Additional file 3: Fig. S1), suggests that overexpression of NTPDase3 may be a hallmark of osteogenic deficiency in Pm women. Figure 1 also shows that NTPDase8 is the least represented ectoenzyme of this series in BM-MSCs from both women groups, strengthening the idea that it might have a limited role in human osteogenesis.

Ecto-5′-nucleotidase/CD73 is expressed by more than 95% of osteoprogenitor cells [36]. Even though we show that ecto-5′-nucleotidase/CD73 immunoreactivity is present in BM-MSCs at all differentiation stages (Fig. 1A; see also Ref [6]), the density of this adenosine-forming enzyme is consistently reduced (by 30–60%) in the cells from Pm women compared to those obtained from younger females (Fig. 1Bv).

### Age-related variation in the global NTPDase activity of human BM-MSCs undergoing osteogenic differentiation

**Fig. 2:**
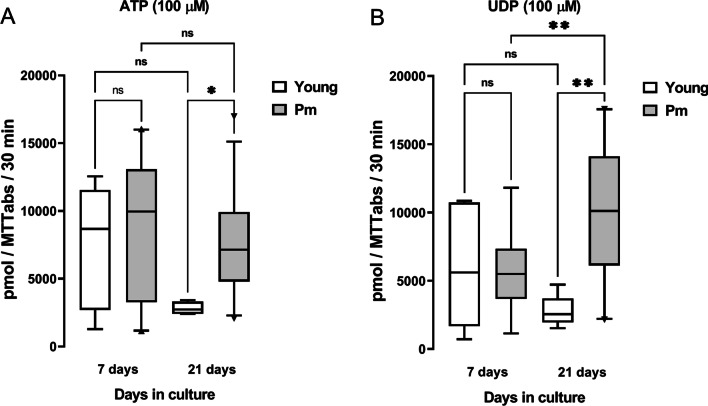
The extracellular catabolism of ATP and UDP is faster in BM-MSCs from Pm women than in cells from younger females. Represented is the total NTPDase activity measured in BM-MSCs (first subculture) from young females and Pm women normalized by the number of viable cells given by the MTT assay when **A** ATP and **B** UDP were used as substrates. Cells were allowed to grow for 7 and 21 days in an osteogenic-inducing medium. Nucleotides (100 µM) were added to the culture medium at zero time. Samples (75 µl) were collected from each well 30 min after incubation with the nucleotides. Each collected sample was analysed by HPLC to measure the amounts of ATP or UDP remaining in the incubation fluid. Boxes and whiskers represent pooled data from three young females (19 ± 6 years old) and twelve Pm women (69 ± 3 years old); each individual was tested in triplicate. *P < 0.05 and **P < 0.01 (one-way ANOVA with Sidák’s multiple comparison test, single pooled variance) represent significant differences

Figure 2 illustrates the global NTPDase activity in osteogenic differentiating BM-MSC cultures from Pm women and younger females normalized by cells growth/viability (MTT assay) using ATP (Fig. 2A) and UDP (Fig. 2B) as substrates (100 µM, for 30 min). Data show that BM-MSCs from Pm women hydrolyze ATP (100 µM) 1.16 and 2.63 times faster than cells from younger females considering culture days 7 and 21, respectively (Fig. 2A). The extracellular hydrolysis of UDP (100 µM) was slower in less differentiated cells (7-day cultures), but it exceeded the rate of ATP (100 µM) catabolism by Pm BM-MSC cultures at culture day 21 (Fig. 2B); at this time point, the total NTPDase activity was 3.64 times faster than that achieved in cells from younger females (Fig. 2B).

### Inhibition of NTPDase3 activity increases the extracellular accumulation of ATP and UDP in osteogenic differentiating BM-MSCs from Pm women

**Fig. 3:**
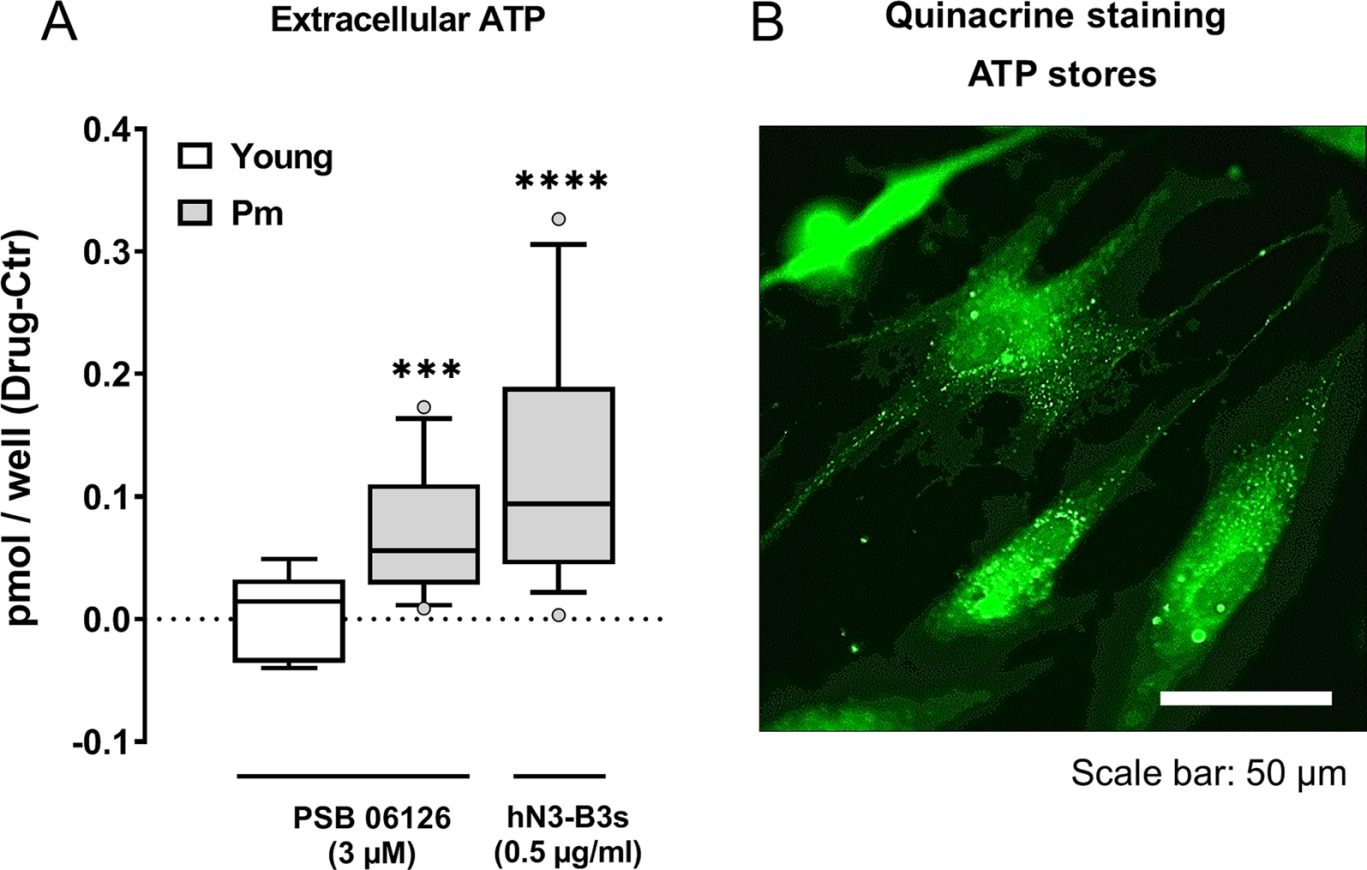
Selective inhibition of NTPDase3 with PSB 06126 (3 µM) or the monoclonal antibody hN3-B3_S_ (0.5 µg/ml) increases the extracellular ATP accumulation in Pm BM-MSCs cultures, but not in those obtained from younger females, when cells were allowed to grow for 7 days in an osteogenic-inducing medium (first subculture). In panel A, ordinates are the difference between endogenously released ATP during 30 min by cells of the same individual incubated in the presence and the absence of NTPDase3 inhibitors (Drug-Ctr, respectively); zero represents the identity between ATP values obtained in the absence and the presence of the inhibitors (horizontal dashed line). The ATP content of the samples was measured using the luciferin-luciferase bioluminescence assay (75 µl/well; see Materials and Methods for details). Boxes and whiskers represent pooled data from three younger females (21 ± 7 years old; ATP release in Ctr: 90 ± 20 fmol/well) and six Pm women (68 ± 5 years old; ATP release in Ctr: 180 ± 40 fmol/well); two to four replicates were performed per individual. ***P < 0.001 and ****P < 0.0001 (Wilcoxon signed rank test for comparing medians with a hypothetical null variation) represent significant differences. Panel B shows that BM-MSCs from a Pm woman undergoing osteogenic differentiation exhibit high amounts of ATP inside intracellular granules stained with quinacrine. The scale bar is 50 µm

So far, we showed that NTPDase3 expression increases over time in cultured BM-MSCs obtained from Pm women (Fig. 1Biii), which correlates with faster kinetics of ATP and UDP breakdown in these cells compared to those originating from younger females (Fig. 2). Next, we questioned whether the age-related upsurge in the expression and/or activity of NTPDase3 contributes to impair the osteogenic commitment of Pm BM-MSCs owed to tonic P2X7 and P2Y_6_ purinoceptors activation by endogenously released ATP and UDP, respectively. Figure 3A shows that the selective inhibition of NTPDase3 with two chemically distinct compounds, the anthraquinone derivative PSB 06126 (3 µM) [37] and the monoclonal antibody hN3-B3_S_ (0.5 µg/ml) [26], significantly increased ATP levels in the incubation medium of Pm BM-MSC cultures, but the same was not observed when cultured cells were obtained from younger females.

To rule out the possibility that loss of the purinergic tone in cultured BM-MSCs from Pm women could be due to the depletion of ATP-containing intracellular reservoirs, we labelled the cells with quinacrine, a fluorescent dye normally staining ATP-containing granules. Figure 3B shows that quinacrine fluorescence exists scattered all over the cytoplasm of cultured Pm BM-MSCs in significant amounts, thus reproducing the staining pattern obtained by other groups in differentiated osteoblasts [15].

**Fig. 4:**
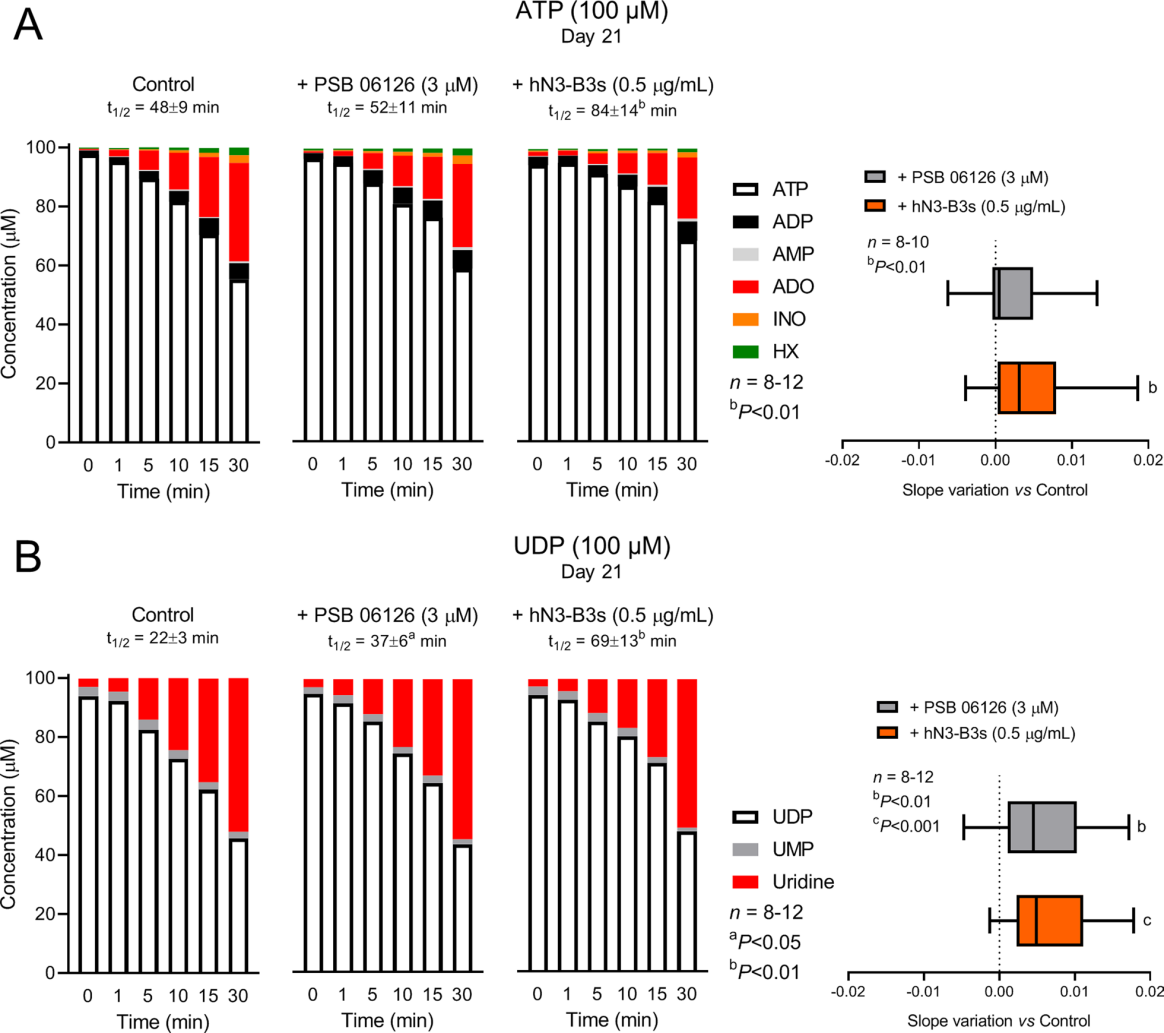
Selective inhibition of NTPDase3 attenuates the extracellular catabolism of **A** ATP and **B** UDP in BM-MSCs from Pm women allowed growing for 21 days in an osteogenic-inducing medium. ATP (100 μM) or UDP (100 μM) were added to the culture medium at time zero either in the absence (left-hand-side panels) or in the presence of PSB 06126 (3 µM; middle panels) or hN3-B3_S_ (0.5 µg/mL; right-hand-side panels). 75-μl samples were collected at the indicated times for HPLC analysis to quantify the substrates ATP or UDP (white bars) and their metabolites, namely ADP (black), AMP (grey), adenosine (ADO, red), inosine (INO, orange), hypoxanthine (HX, green) or UMP (grey) and uridine (red). The calculated half-life time (t_1/2_) for each initial substrate is shown for comparison; ^a^*P* < 0.05 and ^b^*P* < 0.01 (two-tailed unpaired *t*-test) represent significant differences. Right-hand-side box and whiskers graphs represent the effect of PSB 06126 (3 µM) and hN3-B3_S_ (0.5 µg/mL), assessed by comparing the slope variation of ATP or UDP disappearance in the presence of NTPDase3 inhibitors or under control conditions (with no added drugs); zero represents identity between obtained values in the absence and the presence of the inhibitors. Box and whiskers represent pooled data from eight to twelve Pm women (69 ± 3 years old); cells from each individual were tested in triplicate. ^b^*P* < 0.01 and ^c^*P* < 0.001 (one-way ANOVA with Sidák’s multiple comparison test, single pooled variance) represent significant differences

Selective inhibition of NTPDase3 with PSB 06126 (3 µM) and hN3-B3_S_ (0.5 µg/ml) decreased the extracellular catabolism of ATP (100 µM; Fig. 4A) and UDP (100 µM; Fig. 4B) in osteogenic differentiating Pm BM-MSC cultures. While PSB 06126 (3 µM) had only a minor increasing effect on the extracellular ATP (100 µM) half-life, inhibition of NTPDase3 with the monoclonal antibody hN3-B3_S_ (0.5 µg/ml) increased the half-life of this nucleotide by 1.75 times (Fig. 4A). ATP (100 μM) was sequentially metabolized into ADP, AMP, adenosine (ADO), inosine (INO) and hypoxanthine (HX); at the end of the 30-min incubation period, adenosine was the more represented ATP metabolite, but its levels significantly decreased in the presence of NTPDase3 inhibitors (Fig. 4A). Overall, these findings, together with the negligible accumulation of AMP, INO and HX in the cultures, are in keeping with the concept that human BM-MSCs usually exhibit high ecto-5′-nucleotidase/CD73 and low adenosine deaminase (ADA) activities, thus favouring the accumulation of adenosine as the main extracellular ATP metabolite (see e.g. Ref [3]).

Likewise, both PSB 06126 (3 µM) and hN3-B3_S_ (0.5 µg/ml) increased the extracellular half-life of UDP (100 µM) by 1.68 and 3.14 times, respectively (Fig. 4B). Comparing the slope variation of extracellular ATP and UDP disappearance from culture media, data indicate that selective NTPDase3 inhibition was more effective towards inhibition of extracellular UDP catabolism than that of ATP (Fig. 4A and B, right-hand-side panels). Accumulation of UMP was almost negligible throughout the enzymatic assay, which may be explained considering that these cells exhibit significant ecto-5′-nucleotidase/CD73 activity converting very rapidly adenine/uracil monophosphates into their nucleoside metabolites, either adenosine (Fig. 4A) or uridine (Fig. 4B).

### Inhibition of NTPDase3 activity fosters osteogenic differentiation and matrix mineralization of cultured BM-MSCs from Pm women

**Fig. 5:**
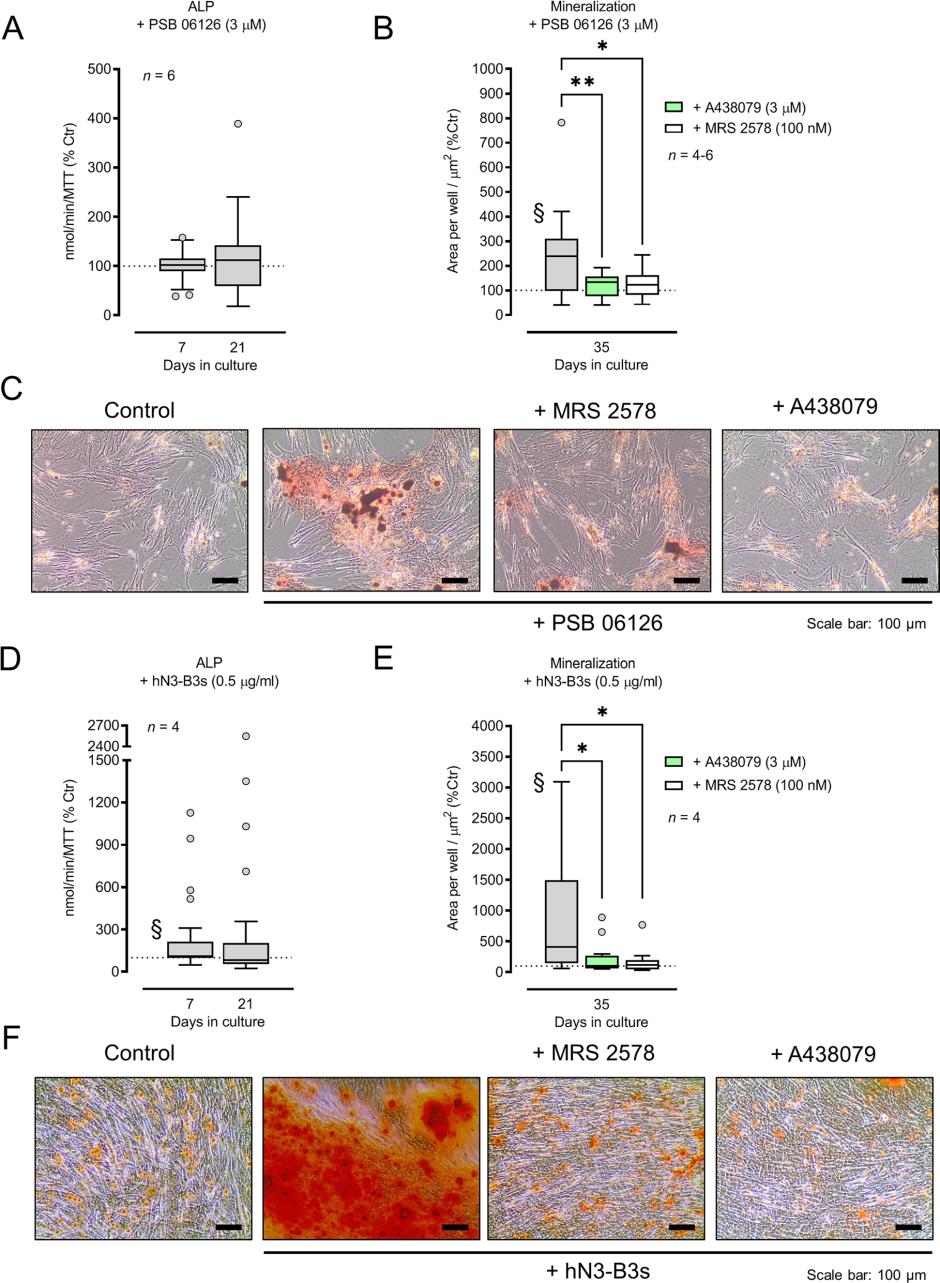
Selective inhibition of NTPDase3 activity with the pharmacological inhibitor, PSB 06126 (3 µM; panels **A**–**C**), or with the monoclonal antibody, hN3-B3_S_ (0.5 µg/ml; panels **D**–**F**), facilitates osteogenic differentiation and mineralization of cultured BM-MSCs from Pm women via the activation of P2X7 and/or P2Y_6_ receptors. Each inhibitor was added to osteogenic-inducing media at culture day 0 and remained throughout the assay, i.e. until day 35. Panels A and D show the per cent variation of the ALP activity of Pm BM-MSC cultures on days 7 and 21 corrected for cells growth/viability (MTT assay) determined in the presence of NTPDase3 inhibitors versus the control situation with no added drugs (100%; 9.6 ± 2.6 and 18.3 ± 4.0 nmol/min/MTT at days 7 and 21, respectively). Panels B and E show the per cent variation of the total mineralized area of Pm BM-MSC at culture day 35 in the presence of NTPDase3 inhibitors compared to the control situation with no added drugs (100%; 10,079.2 ± 4210.5 µm^2^). ^§^*P* < 0.05 (Wilcoxon signed rank test for comparing medians with a hypothetical null variation, 100%) represent significant differences. Involvement of P2X7 and P2Y_6_ purinoceptors activation in extracellular matrix mineralization was confirmed using selective antagonists, A438079 (3 µM) and MRS 2578 (100 nM), respectively, which were added to culture media synchronously to NTPDase3 inhibitors. Panels C and F show Alizarin Red staining indicating extracellular matrix mineralization and bone nodule formation of BM-MSC cultures (red-brownish spots) in two Pm women; the scale bar is 100 µm. Boxes and whiskers represent pooled data from four to six Pm women (70 ± 3 years old); three to eight replicas were made per individual experiment. *P < 0.05 and **P < 0.01 (one-way ANOVA with Sidák’s multiple comparison test, single pooled variance) represent significant differences

Selective inhibition of NTPDase3 with the monoclonal antibody hN3-B3_S_ (0.5 µg/ml; Fig. 5D), but not with the anthraquinone derivative PSB 06126 (3 µM; Fig. 5A), increased the ALP activity of osteogenic differentiating Pm BM-MSCs by 237 ± 63% and 329 ± 129% above control levels at culture days 7 and 21, respectively. None of these compounds changed (*P* > 0.05) cells growth/viability predicted by the MTT assay obtained in the presence of PSB 06126 (3 µM; 0.22 ± 0.02 vs. control of 0.23 ± 0.02 at culture day 7, and 0.56 ± 0.07 vs. control of 0.63 ± 0.08 at culture day 21) or of hN3-B3_S_ (0.5 µg/ml; 0.33 ± 0.06 vs control of 0.34 ± 0.06 at culture day 7, and 0.63 ± 0.09 vs control of 0.62 ± 0.09 at culture day 21). Notwithstanding this disparity, both PSB 06126 (3 µM) and hN3-B3_S_ (0.5 µg/ml), significantly (*P* < 0.05) increased matrix mineralization on Pm BM-MSC culture day 35 by 243 ± 35% (Fig. 5B) and 842 ± 282% (Fig. 5E) above control values, respectively; typical Alizarin Red histoenzymatic culture staining is shown in panels C and F of Fig. 5, respectively.

Activation of P2X7 and P2Y_6_ purinoceptors are most likely implicated in the promotor effects of PSB 06126 (3 µM) and hN3-B3_S_ (0.5 µg/ml) on matrix mineralization of Pm BM-MSC cultures [5, 6, 38], considering that these effects were prevented by A438079 (3 µM; Fig. 5B and C) and by MRS 2578 (100 nM; Fig. 5E and F), which selectively antagonize P2X7 and P2Y_6_ receptors, respectively. On their own, these two purinoceptor antagonists did not change the total mineralized area of the cultures. These findings indicate that endogenously produced adenine and uracil nucleotides by Pm BM-MSCs might not reach high enough concentrations required to activate P2X7 and P2Y_6_ receptors unless the NTPDase3 activity is inhibited to recapitulate the lack of expression/activity of this enzyme (see Fig. 1 and 2) and the mineralization potential of BM-MSCs from younger females [5, 6].

### Transient NTPDase3 gene silencing using a short hairpin RNA rescues the osteogenic differentiation potential of cultured BM-MSCs from Pm women

**Fig. 6:**
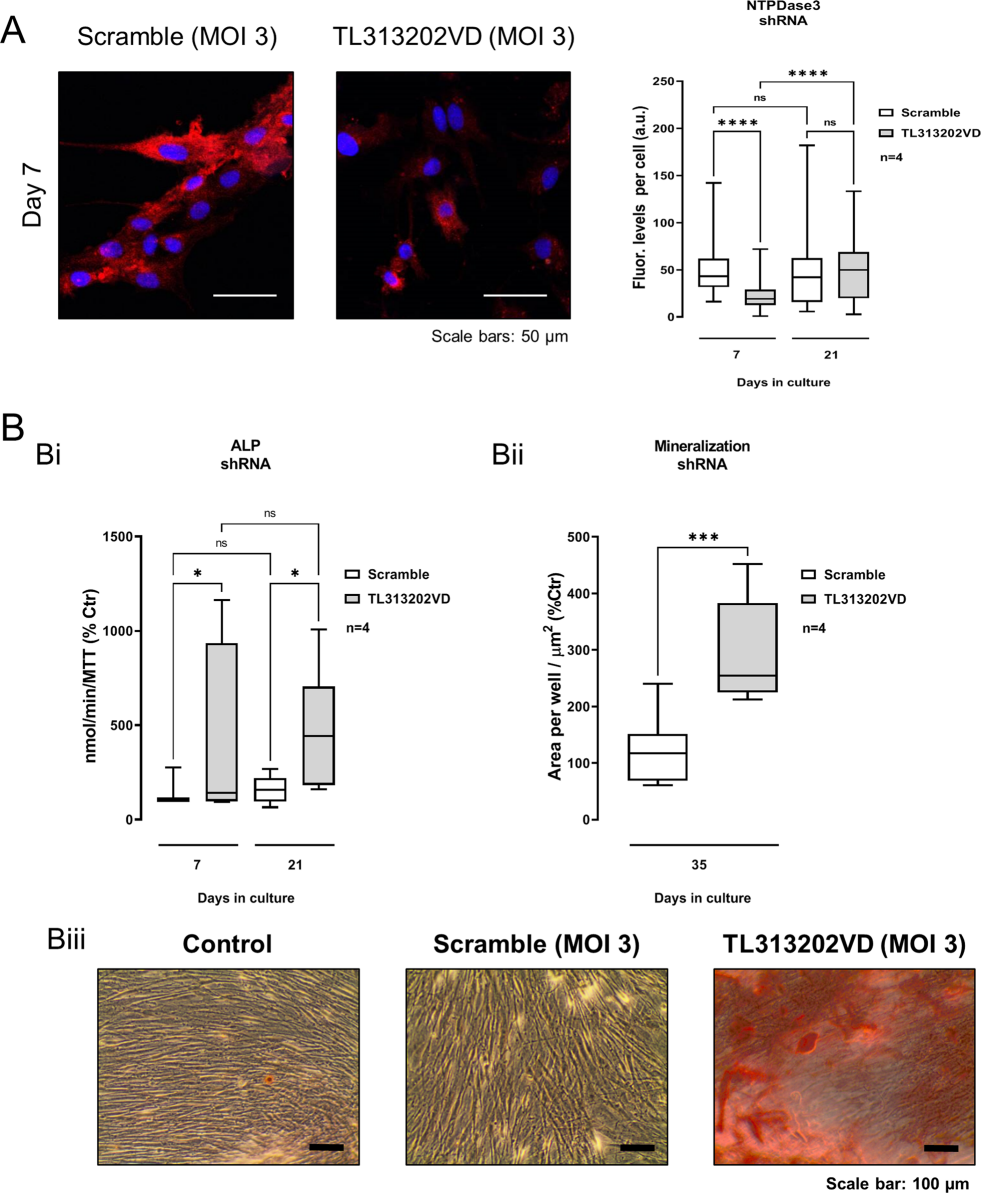
NTPDase3 gene silencing using lenti-shRNA promotes mineralization of Pm BM-MSC cultures allowed to grow for 35 days in an osteogenic-inducing medium. Cells were exposed for 24 h to a lenti-shRNA encoding to a sequence (TL313202VD) designed to silence NTPDase3 or to a scramble sequence applied at multiplicities of infection 3 (MOI 3). Confocal microscopy images shown in panel A confirm that NTPDase3 immunoreactivity transiently decreases 7-days after exposure of Pm BM-MSC cultures to a lenti-shRNA encoding to the TL313202VD sequence, but not when the cells were treated with the scramble sequence. Blue dots represent nuclei stained with DAPI. Experiments were performed in parallel keeping unaltered the settings of the confocal microscope throughout the documentation procedure (see Materials and Methods). The scale bar is 50 µm. Panel Bi shows the per cent variation of the ALP activity of Pm BM-MSC cultures on days 7 and 21 corrected for cells growth/viability (MTT assay) obtained after treatment with the lenti-shRNA encoding to TL313202VD or the scramble sequence (MOI 3) (100%; 0.6 ± 0.1 and 7.1 ± 3.7 nmol/min/MTT at days 7 and 21, respectively). Panel Bii shows the per cent variation of the total mineralized area of Pm BM-MSCs at culture day 35 after treatment with the lenti-shRNA encoding to TL313202VD or the scramble sequence (MOI 3) (100%; 13,714.1 ± 3657.6 µm^2^). Panel Biii shows the Alizarin Red staining denoting extracellular matrix mineralization and bone nodule formation of BM-MSC cultures (red-brownish spots) in a Pm woman; the scale bar is 50 µm. Boxes and whiskers represent pooled data from four Pm women (69 ± 7 years old); three to four replicas were made per individual. In panel Bi, *P < 0.05 (one-way ANOVA with Sidák’s multiple comparison test, single pooled variance) represents significant differences; in panel Bii, ***P < 0.01 (two-tailed unpaired *t*-test) represent significant differences

To avoid the negative impact on osteogenic differentiation and matrix mineralization of NTPDase3 overexpression in BM-MSCs from Pm women, we infected the cells with a series of lentiviruses carrying information to short hairpin RNAs (shRNAs) designed to silence NTPDase3 gene expression. The most effective sequence (TL313202VD from ORIGENE) was selected among four blocking sequences tested together with a scramble sequence (negative control) unable to associate with the target mRNA (see Materials and Methods; validation experiments are shown in Additional file 1: Fig. S2). Treatment of Pm BM-MSC cultures with the lenti-shRNA encoding to the TL313202VD sequence at a multiplicity of infection 3 (MOI 3) transiently decreased (*P* < 0.05) the NTPDase3 immunoreactivity of the cells at culture day 7 (23 ± 1 a.u.) compared to the situation where the scramble sequence was used (51 ± 2 a.u.); full recovery of NTPDase3 protein amounts was observed at culture day 21 (Fig. 6A).

Even though NTPDase3 gene silencing transiently decreased protein production for only a week under these experimental conditions, it had an outstanding effect to promote osteogenic commitment (increase in ALP activity at culture days 7 and 21; Fig. 6Bi) and matrix mineralization (Alizarin Red bone nodules increased by 297 ± 32% at day 35; Fig. 6Bii and 6Biii) of cultured BM-MSCs from Pm women, without significantly modifying cells growth/viability (MTT assay; data not shown).

### Inhibition of NTPDase3 expression/activity rehabilitates age-dependent deficits of the osteogenic transcription factor, Osterix, in cultured Pm BM-MSCs

**Fig. 7:**
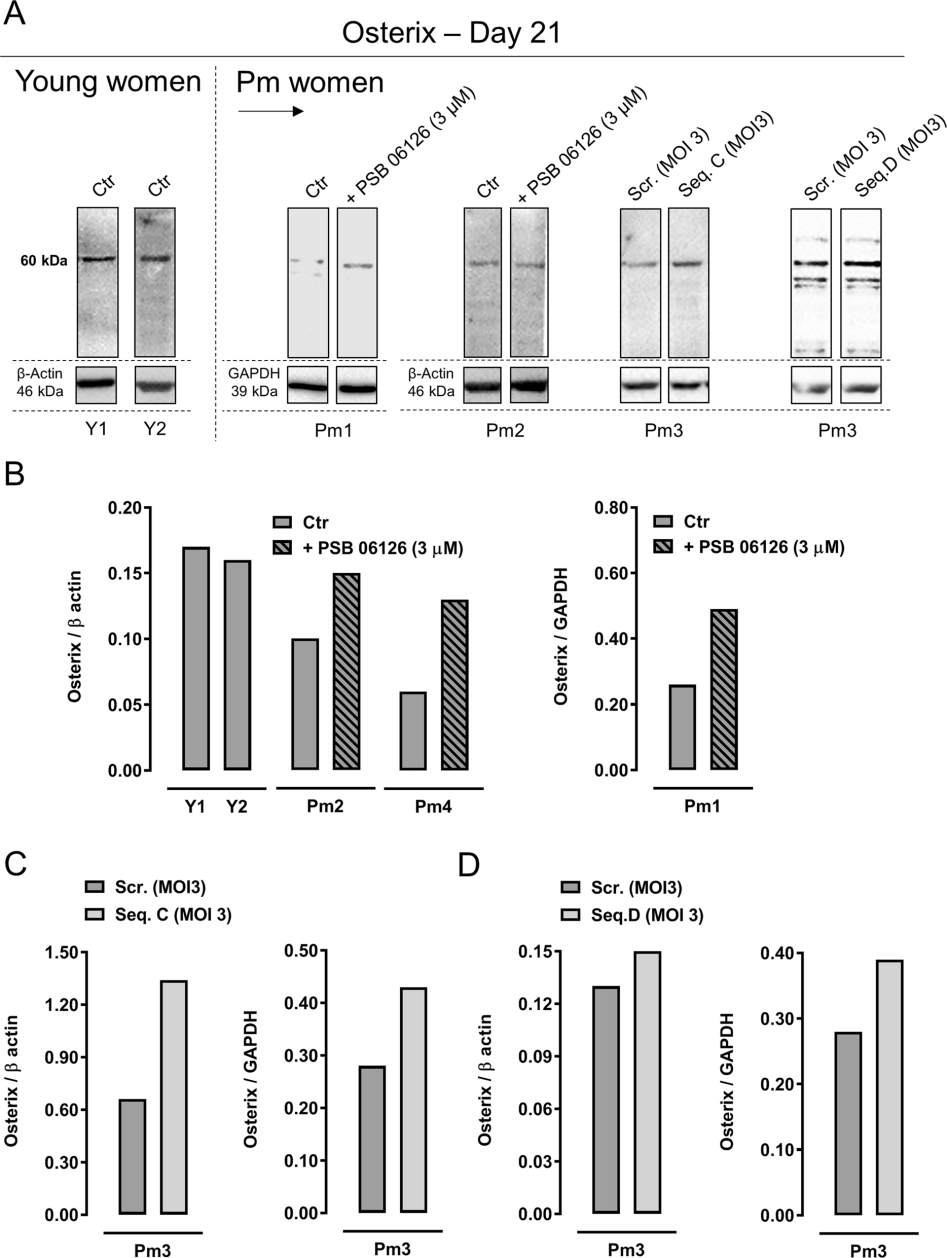
Inhibition or silencing of NTPDase3 increases the amount of osteogenic transcription factor Osterix in 21-day BM-MSC cultures (first subculture) obtained from Pm women to the levels found in younger females. In panel A, shown are typical immunoblots stained for Osterix (52–61 kDa) transcription factor in BM-MSCs from two young (Y1 and 2) and three Pm (Pm1, 2 and 3) women cultured in an osteogenic-inducing medium (i) in the absence or the presence of the NTPDase3 inhibitor, PSB 06126 (3 µM), or (ii) after NTPDase3 gene silencing with lenti-shRNAs encoding for TL313202VD (Seq D; MOI 3) and TL313202VC (Seq C; MOI 3) inhibitory vs. control scramble sequences; either β-Actin (46 kDa) or GAPDH (39 kDa) protein amounts were used as house-keeping gene product standards for normalization purposes. Uncropped full-length gel blots can be found in Additional file 4: Fig. S4. Panel B shows the relative expression of Osterix in BM-MSCs from young females and Pm women submitted or not to NTPDase inhibition with PSB 06126 (3 µM) or to gene silencing with lenti-shRNAs encoding for TL313202VD (Seq D; MOI 3) and TL313202VC (Seq C; MOI 3). Each bar corresponds to the results from a single patient, thus reflecting some inter-individual variability

Osterix is a zinc finger-containing transcription factor that is essential for osteoblast differentiation and bone formation [39]. Osterix deficiency has been associated with age-related osteoporosis (reviewed in [40]). The immunoblots depicted in Fig. 7A show that 21-day BM-MSC cultures from two younger females express higher amounts of Osterix normalized by the housekeeping protein β-actin than the cells from Pm women grown under similar experimental conditions (Fig. 7B).

Pharmacological inhibition of NTPDase3 enzymatic activity of Pm BM-MSCs with PSB 06126 (3 µM) recapitulates the Osterix/β-actin ratio to levels observed in younger females (Fig. 7B). A similar effect was also observed using a different protein normalizer, GAPDH, in cells from a distinct Pm woman (Fig. 7B).

Similarly, NTPDase3 gene silencing in Pm BM-MSCs using lenti-shRNAs encoding to TL313202VC (Seq C; MOI 3; Fig. 7C) and TL313202VD (Seq D; MOI 3; Fig. 7D) sequences also increased the relative amount of the Osterix transcription factor independently of the normalizer protein used, β-actin or GAPDH. Uncropped full-length gel blots may be found in Additional file 4: Fig. S4. Altogether, these findings suggest that curbing the expression and/or activity of the nucleotide metabolizing enzyme, NTPDase3, may recapitulate the osteogenic potential of Pm BM-MSCs to the levels observed in younger females.

## Discussion

Previous accounts from our group suggest that the osteogenic differentiation potential of BM-MSCs is compromised in Pm women compared to that of younger females [5, 6]; this situation may be in part attributed to impairment of the P2X7 and P2Y_6_ purinoceptors tonic activation by released adenine and uracil nucleotides, such as ATP and UDP, respectively [5, 6]; for a review, see e.g. Ref [7]. Here, we provide new evidence that the purinergic signalling deficiency in Pm women is caused by outstanding hydrolysis of extracellular nucleotides due to NTPDase3 overexpression, which turns out to be the dominant ectonucleotidase subtype at this age and gender group. This contrasts with the minute amounts of the NTPDase3 enzyme found in osteogenic differentiating BM-MSCs of younger females and age-matched males. More importantly, our data show for the first time that transient NTPDase3 gene silencing with a lenti-shRNA and/or inhibition of its enzymatic activity with the anthraquinone derivative PSB 06126 [37] or the monoclonal antibody hN3-B3_S_ [26] rescues the P2X7 and P2Y_6_ receptors-induced osteogenic commitment of Pm BM-MSC. Consequently, this results in extensive matrix mineralization of Pm BM-MSC cultures to the levels found in younger females. These findings lead us to propose that inhibition of NTPDase3 expression and/or activity may be a novel therapeutic strategy to rehabilitate the osteogenic potential of senescent BM-MSCs that is required to increase bone formation in Pm women.

Human osteoprogenitor MSCs and osteoblast-like cells constitutively release ATP without cell damage; the estimated intracellular ATP levels are between 2 and 5 mM [11, 14, 41–43], most of which can be easily identified in vesicular/granular organelles stained with fluorescent dyes, like quinacrine [15]. Here, we found that quinacrine-stained ATP-containing granules were scattered abundantly in the cytoplasm of Pm BM-MSCs, meaning that loss of intracellular ATP reservoirs does not seem to account significantly for age-dependent purinergic signalling deficits and, thus, the shortage of the osteogenic commitment of Pm BM-MSCs.

Like ATP, the ability of various cell types (e.g. murine airway epithelial cells, human cardiomyocytes) to release UTP has been directly confirmed; using a laborious enzymatic assay, the release of UTP is estimated to be approximately 10 to 30% of all detected nucleotides including its breakdown products, like UDP [44, 45]. Likewise, UDP-sugars, like the P2Y_14_ receptor activator UDP-glucose, may be released from cells together with ATP under certain experimental conditions [44, 46, 47]. UDP itself is an end product of glycogen synthesis that may be released into the extracellular fluid [48, 49]. Yet, the extracellular accumulation of uracil nucleotides, such as UTP and UDP, does not necessarily depend on their outflow from intracellular pools via dedicated transport systems; these nucleotides may accumulate in the extracellular fluid as a consequence of the metabolism (hydrolysis or synthesis) of nucleotide precursors that are directly released by cells under various physiological and pathological conditions [50–54].

There are two main mechanisms underlying the non-lytic release of nucleotides from cells: (a) exocytotic release, specifically concentrated within secretory granules or vesicles, and (b) controlled release of cytosolic nucleotides via intrinsic plasma membrane channels or pores, which includes ABC transporters, connexin hemichannels, voltage-dependent anion channels and the P2X7 receptor channel itself [11, 15, 55]; reviewed in [56]. Increasing evidence demonstrates that all stimuli that foster bone formation and accelerated fracture healing (e.g. mechanical loading, low-intensity pulsed ultrasound, 1,25(OH)_2_ vitamin D3, biphosphonates) favour ATP release from osteoblast-like cells [8–13], which levels depend on the differentiation status of the cells [15]; using rat osteoblasts, these authors demonstrated that mature bone-forming cells release up to sevenfold more ATP than highly-proliferative immature cells. In this study, we failed to demonstrate any tonic activity of the P2X7 receptor in Pm BM-MSCs using its selective antagonist, A438079, which was applied in a concentration (3 µM) that almost prevented the ALP activity in cells from younger females grown under similar experimental conditions [5], and the same occurred regarding the P2Y_6_ receptor [6].

Considering that no differences were observed in the expression of osteogenic-inducing P2X7 and P2Y_6_ receptors between young and aged women groups [5, 6], we hypothesized that deficient activity of these two receptors may exist because their endogenous ligands (ATP and UDP, respectively) cannot reach high enough levels in cells from Pm women unless NTPDases (namely NTPDase3) expression and/or activity are inhibited. NTPDase3 is overexpressed in Pm BM-MSCs compared to the cells originating from younger females and age-matched males, thus contributing to accelerating the breakdown of ATP and UDP released in the proximity of ionotropic P2X7 and metabotropic P2Y_6_ receptors. Moreover, we proved that (i) endogenously released ATP accumulates in culture media when Pm BM-MSCs were incubated with the two selective NTPDase3 inhibitors, PSB 06126 and hN3-B3_S_, and that (ii) the kinetics of the extracellular ATP and UDP catabolism was delayed in the presence of either of these two NTPDase3 inhibitors. Moreover, transient NTPDase3 gene silencing with the lenti–shRNA encoding for the TL313202VD sequence and enzyme inhibition with PSB 06126, both rescued the expression of the osteogenic transcription factor Osterix, the ALP activity and matrix mineralization of Pm BM-MSC cultures grown in osteogenic-inducing media to the levels observed in cells from younger females. Osterix is known to trigger BM-MSCs differentiation into mature osteoblasts leading to the subsequent production of extracellular matrix proteins involved in mineralization, namely bone sialoprotein, osteocalcin and osteopontin, making it an ideal primary marker of the osteogenic commitment of these cells (see e.g. Ref [39]).

Species and cell maturation differences in the expression and activity of certain nucleotide metabolizing enzymes, like NTPDases, [6] may at least partially explain divergent findings in the literature regarding the involvement of P2 purinoceptors in osteogenesis (reviewed in [7]). The age-dependent extent of the extracellular catabolism of most commonly used P2X7 receptor agonists, like BzATP, may also account for these differences if one does not take into account the expression and activity of NTPDases [5]. Likewise, the preferential P2Y_6_ receptor activation *vis a vis* other pyrimidine-sensitive P2 receptor subtypes may be explained because distinctively from ATP catabolism that can either yield ADP or AMP, all membrane-bound NTPDases dephosphorylate UTP with a transient formation of the diphosphate metabolite, UDP [57]. One cannot however discount the fact that P2Y_2_ and P2Y_4_ receptors recognizing both ATP and UTP have also been localized in human bone-forming cells [18, 20]. However, controversy still exists regarding the fact that these receptors have been involved in the inhibition of bone mineralization, particularly when nucleotides were used in low concentrations [19, 21].

In contrast to data obtained regarding the kinetics of ATP catabolism, which mostly depends on the number of viable cells at a given cell maturation stage (Fig. 2A), normalization of BM-MSCs' ability to hydrolyze UDP per viable cell (MTT assay) showed that the net enzymatic activity of Pm BM-MSCs, but not those from younger females, substantially increases as cultures progress from day 7 to 21 (Fig. 2B). Since the number of viable cells did not increase proportionally in the same period, the results suggest that mature BM-MSCs from Pm women exhibit higher NTPDase activity implicated in the extracellular inactivation of UDP. The dominant presence of NTPDase3 in differentiated Pm BM-MSCs and its low expression in cells from younger females may give a good explanation for the discrepancy between the two age groups concerning the ability to hydrolyze UDP (and ATP) with a direct impact on the osteogenic commitment of the cells.

BM-MSCs from Pm women and younger females express relatively high amounts of NTPDase1 and 2 (see Ref. [6]). NTPDase1 (nucleotide diphosphohydrolase or apyrase) is expected to terminate the actions of both ATP and UDP on P2X7 and P2Y_6_ receptors, respectively, by their conversion directly into inactive nucleotide monophosphate derivatives (AMP and UMP). Conversely, the presence of NTPDase2 (a preferential nucleotide triphosphatase) may promote the accumulation of nucleotide diphosphates, like UDP, and subsequent P2Y_6_ receptors activation [6]. The virtual absence of NTPDase8 from BM-MSC membranes of both women groups suggests that this enzyme does not play a role in the catabolism of extracellular adenine and uracil nucleotides and, thus, in the osteogenic commitment of these cells.

The decreased osteogenic ability of BM-MSCs from Pm women as a consequence of the presence of high NTPDase amounts converting ATP into AMP could be partially compensated if subsequent dephosphorylation of AMP into adenosine by ecto-5′-nucleotidase/CD73 is endorsed to allow activation of osteogenic-inducing P1 receptors [3]. Regrettably, the density of this adenosine-forming enzyme is diminished in BM-MSCs from Pm women compared to that found in the cells of younger females. These findings comply with those obtained in animals with osteoporotic bone loss [58] also considering that ecto-5′-nucleotidase/CD73 is expressed in more than 95% of the cells exhibiting osteogenic capability [36]. Among the adenosine-sensitive P1 receptors, the most abundant A_2B_ receptor subtype has been implicated in the differentiation of BM-MSCs from Pm women, which action is normally balanced through the activation of coexisting A_1_ or A_2A_ receptors setting whether osteoprogenitor cells are driven into proliferation or differentiation [3]. Despite that adenosine formation was detected as a consequence of the extracellular ATP hydrolysis in mature Pm BM-MSCs cultured in the presence of NTPDase3 inhibitors, PSB 06126 and hN3-B3_S_ (see Fig. 4), rescuing of matrix mineralization under such conditions was fully prevented by selective blockage of P2X7 and P2Y_6_ receptors, ruling out the putative contribution of P1 adenosine receptors in this context.

Interestingly, the monoclonal antibody, hN3-B3_S_, proved to be more potent than the pharmacological NTPDase3 inhibitor, PSB 06126, resulting in powerful increases in P2X7 and P2Y_6_ receptor-operated osteogenic differentiation and matrix mineralization of Pm BM-MSC cultures. Anthraquinone derivatives, such as PSB 06126, inhibit NTPDase isoenzymes via multiple mechanisms depending on the chemical nature of substituent groups, NTPDase enzyme subtypes and animal species [37, 59]. Inhibition of NTPDases by monoclonal antibodies seems to be more consistent and does not vary with the concentration of substrates. This suggests that enzyme inhibition by monoclonal antibodies does not depend on the steric hindrance of the substrate for the active site, but may be due to the restriction of enzyme lobe movements or deformation of the active site [26]. Moreover, monoclonal antibodies may have additional benefits, including fewer off-target adverse effects, lesser drug-drug interactions, higher specificity, and potentially increased efficacy through targeted therapy [60].

However, it is surprising that albeit NTPDase3 gene silencing with the lenti–shRNA was curtailed 7 days after infection of the cells, this procedure was sufficient to increase Osterix transcription, ALP activity and matrix mineralization of Pm BM-MSCs to levels comparable to those found in younger females. The exact trigger and the timing associated with age-related expression of NTPDase3 in Pm BM-MSCs remains uncertain. Data provided in Additional file 3: Fig. S1 show that NTPDase3 immunoreactivity is virtually absent in cultured BM-MSCs from a man requiring bone engraftment due to traumatic fracture, as well as from an older male undergoing total hip replacement due to degenerative osteoarthrosis, besides the lack of NTPDase3 in differentiating cells from young females. One may speculate about the relevance of oestrogen deficiency as a trigger concerning bone loss linked to NTPDase3 overexpression and P2 purinoceptors activation impairment in Pm women, an aspect worth investigating in a near future. Other putative mechanisms involved in the up-regulation of ectonucleotidase enzymes, like those comprising the hypoxia-inducible factor-1α [61–66] and microRNAs [67–69], are also worth pursuing in this context.

## Conclusion

Data show that deficits in the osteogenic differentiation commitment of BM-MSCs may be due to overexpression of the NTPDase3 enzyme leading to excessive extracellular adenine and uracil nucleotides breakdown in bone niches of Pm women. We provide here compelling evidence that the osteogenic potential of these cells can be rehabilitated by NTPDase3 gene silencing (with a lenti-shRNA) or by inhibiting its enzymatic activity with small molecules, like the anthraquinone PSB 06126, or with highly-specific monoclonal antibodies, like hN3-B3_S_. The aforementioned gaps in our knowledge do not invalidate the putative clinical impact of our findings concerning the therapeutic benefit of NTPDase3 gene silencing and/or enzymatic inhibition to increase bone formation in Pm women, as well as in other situations where bone destruction exceeds bone formation (e.g. osteoporosis, rheumatoid arthritis, osteogenesis imperfecta, fracture mal-union). The literature is relatively scarce concerning NTPDase3 expression and function in other tissues. NTPDase3 is relatively abundant in pancreatic β-cells where it may function as a regulator of glucose-induced insulin secretion [70, 71]. It was also found in hypocretin-1/orexin-A positive axon terminals of hypothalamic regions controlling feeding, circadian rhythm and reproduction behaviour [72, 73]. Obesity, metabolic syndrome and osteoporosis are major global health problems with increasing prevalence. The interactions between these conditions turn out to be more complex than previously anticipated, opening new opportunities for a central role of mechanisms involving the purinergic cascade in all these pathological conditions. Finally, the marked differences found between young vs. Pm women (and age-matched males) may suggest NTPDase3 overexpression being considered a novel clinical surrogate for the identification of osteogenic differentiation impairment of BM-MSCs among Pm women with high osteoporotic fracture risk. Whether this feature also applies to MSCs originating from other sources easier to deal with in clinical settings, requires further investigations.

## Supplementary Information

**Additional file 1**. Fig. S2 Efficacy of NTPDase3 gene silencing in 7-day BM-MSC cultures (first subculture) from a Pm woman undergoing osteogenic differentiation infected with several lenti-shRNAs encoding for four inhibitory and one scramble (negative control) sequences at increasing multiplicities of infection (MOI: 1, 3, 10). Left-hand-side micrographs show positive NTPDase3 immunostaining (red) in non-treated cells and the corresponding negative control (where no primary antibody was added). The positive transduction marker GFP (green) is detectable in some of the cells; blue dots represent nuclei stained with DAPI. All experiments were performed in parallel keeping unaltered the settings of the confocal microscope throughout the procedure (see Materials and Methods). The scale bar is 50 µm

**Additional file 2**. Fig. S3 Negative controls of immunofluorescence staining using human BM-MSC cultures allowed growing for 7 days in an osteogenic-inducing medium (first subculture). Panel A, shown is the immunofluorescence staining detected in cells incubated with the secondary antibodies, but where primary antibodies were omitted. Data in panel B show that no immunoreactivity was obtained when primary antibodies were substituted by the corresponding IgG antibodies or by pre-immune sera followed by respective secondary antibodies (anti-mouse, anti-rabbit or anti-guinea pig). Blue dots represent nuclei stained with DAPI contained in the VectaShield mounting medium. The scale bar is 50 µm. gp, guinea pig; m, mouse; rb, rabbit

**Additional file 3**. Fig. S1 Immunocytochemical detection of NTPDase1, -2, -3, -8 and ecto-5’-nucleotidase (Ecto 5’-NT) in cultured BM-MSCs (first subculture) from young (Y) and older (O) males, which were allowed to grow for 7 and 21 days in an osteogenic-inducing medium. Blue dots represent nuclei stained with DAPI. These experiments were performed in parallel to those using women's samples shown in Figure 1 while keeping unaltered the settings of the confocal microscope throughout the experimental procedure (see Materials and Methods). The scale bar is 50 µm

**Additional file 4**. Fig. S4 Uncropped full-length Western-blot gels depicted in Figure 7A (WB1-5). Shown are typical immunoblots stained for Osterix (~60 kDa) transcription factor in BM-MSCs from two young (Y1 and 2) and three Pm (Pm1, 2 and 3) women cultured in an osteogenic-inducing medium (i) in the absence or the presence of the NTPDase3 inhibitor, PSB 06126 (3 µM), or (ii) after NTPDase3 gene silencing with lenti-shRNAs encoding for TL313202VD (Seq D; MOI 3) and TL313202VC (Seq C; MOI 3) inhibitory vs. control scramble sequences; either β-Actin (46 kDa) or GAPDH (39 kDa) protein amounts were used as house-keeping gene product standards for normalization purposes. Dashed boxes indicate where blots were cropped for comparison purposes.

## Data Availability

The data that support the findings of this study are available from the corresponding author upon reasonable request.

## Abbreviations

A.u.: Arbitrary units
A438079: 3-[[5-(2,3-Dichlorophenyl)-1H-tetrazol-1-yl]methyl]pyridine hydrochloride
ADA: Adenosine deaminase
ADO: Adenosine
Alizarin red S: 3,4-Dihydroxy-9,10-dioxo-2-anthracenesulfonic acid sodium salt
ALP: Alkaline phosphatase
BM-MSCs: Bone marrow-derived mesenchymal stromal cells
GAPDH: Glyceraldehyde-3-phosphate dehydrogenase
HPLC: High-performance liquid chromatography
Hx: Hypoxanthine
INO: Inosine
LDH: Lactate dehydrogenase
Lenti-shRNA: Lentiviral short hairpin RNA
MRS 2578: N,N′′-1,4-butanediylbis[N′-(3-isothiocyanatophenyl) thiourea
MTT: 3-[4,5-Dimethylthiazol-2-yl]-2,5-diphenyltetrasodium bromide
PBS: Phosphate-buffered saline
Pm: Post-menopausal
PNP: *p*-Nitrophenyl phosphate
PSB 06126: 1-Amino-4-(1-naphthyl)aminoanthraquinone-2-sulfonic acid sodium salt
ROI: Regions of interest
SDS-PAGE: Sodium dodecyl sulphate–polyacrylamide gel electrophoresis
shRNA: Short hairpin RNA
α-MEM: α-Minimal essential medium
