# Supplementary figures and images for: Silencing NTPDase3 activity rehabilitates the osteogenic commitment of post-menopausal stem cell bone progenitors
Source: Stem Cell Res Ther. 2023 Apr 19;14:97. doi: 10.1186/s13287-023-03315-6 (PMC10116749; doi:10.1186/s13287-023-03315-6)

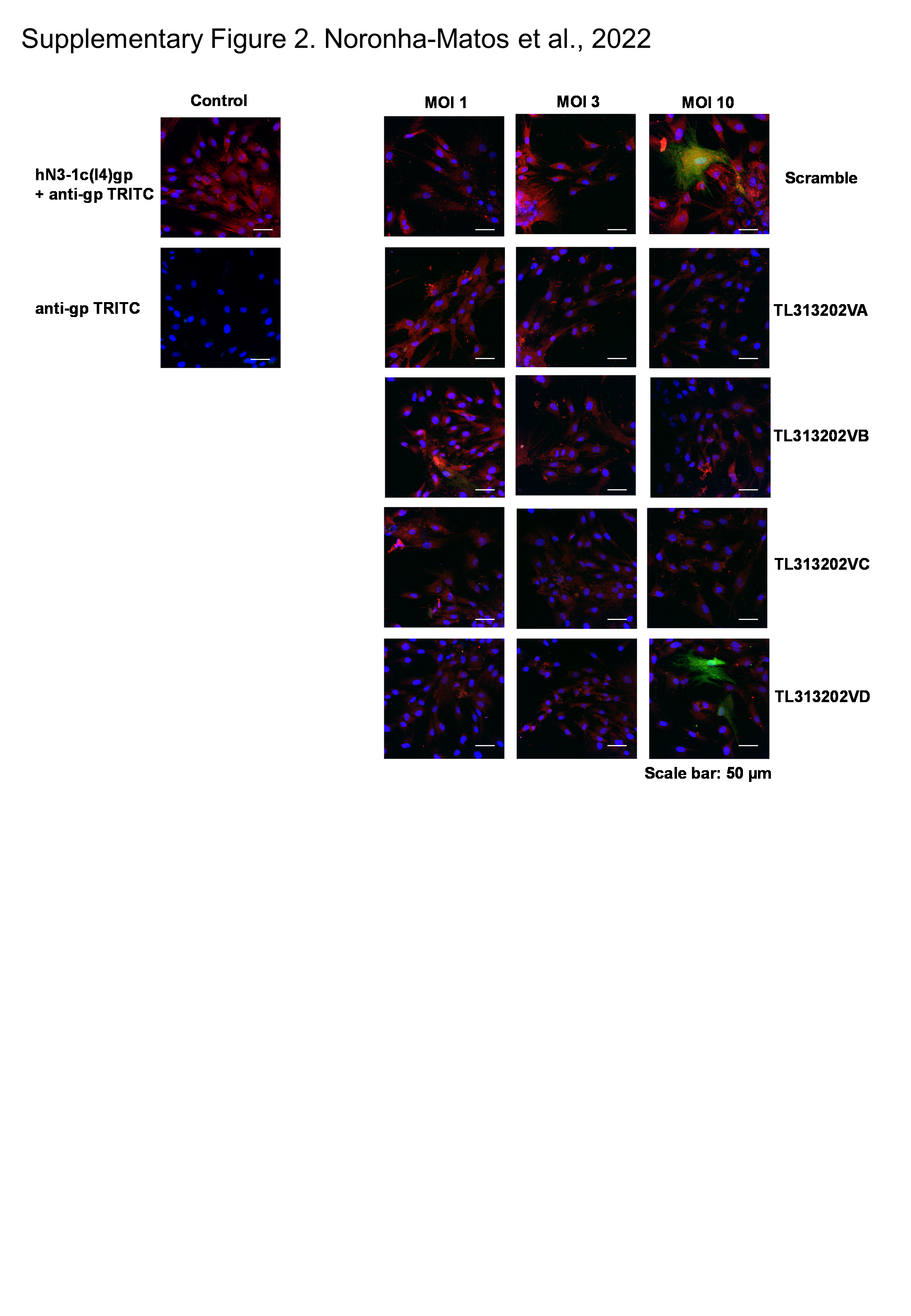

Supplement: Supplementary file 1 — Additional file 1. Fig. S2 Efficacy of NTPDase3 gene silencing in 7-day BM-MSC cultures (first subculture) from a Pm woman undergoing osteogenic differentiation infected with several lenti-shRNAs encoding for four inhibitory and one scramble (negative control) sequences at increasing multiplicities of infection (MOI: 1, 3, 10). Left-hand-side micrographs show positive NTPDase3 immunostaining (red) in non-treated cells and the corresponding negative control (where no primary antibody was added). The positive transduction marker GFP (green) is detectable in some of the cells; blue dots represent nuclei stained with DAPI. All experiments were performed in parallel keeping unaltered the settings of the confocal microscope throughout the procedure (see Materials and Methods). The scale bar is 50 µm [file 13287_2023_3315_MOESM1_ESM.tif]

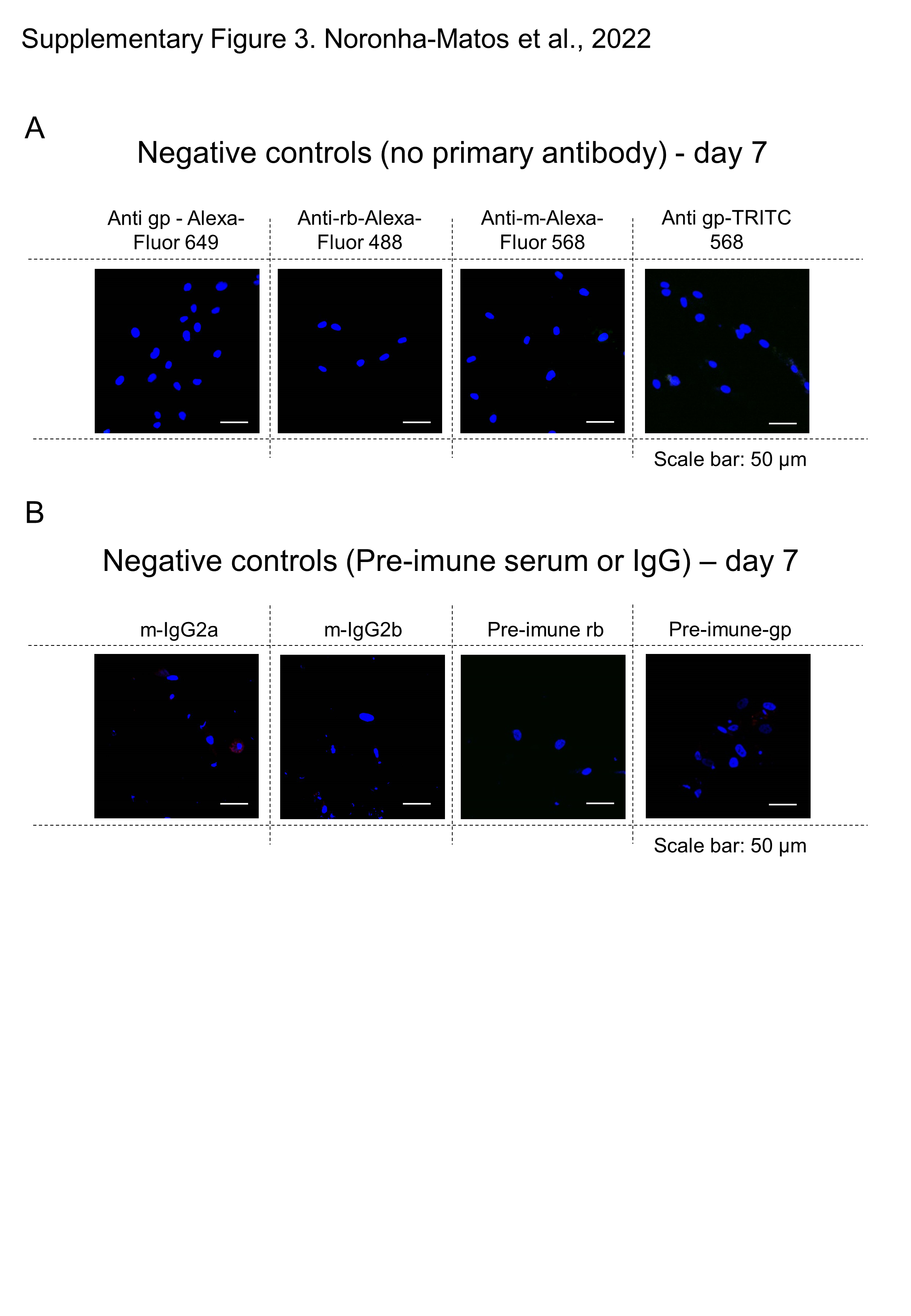

Supplement: Supplementary file 2 — Additional file 2. Fig. S3 Negative controls of immunofluorescence staining using human BM-MSC cultures allowed growing for 7 days in an osteogenic-inducing medium (first subculture). Panel A, shown is the immunofluorescence staining detected in cells incubated with the secondary antibodies, but where primary antibodies were omitted. Data in panel B show that no immunoreactivity was obtained when primary antibodies were substituted by the corresponding IgG antibodies or by pre-immune sera followed by respective secondary antibodies (anti-mouse, anti-rabbit or anti-guinea pig). Blue dots represent nuclei stained with DAPI contained in the VectaShield mounting medium. The scale bar is 50 µm. gp, guinea pig; m, mouse; rb, rabbit [file 13287_2023_3315_MOESM2_ESM.tif]

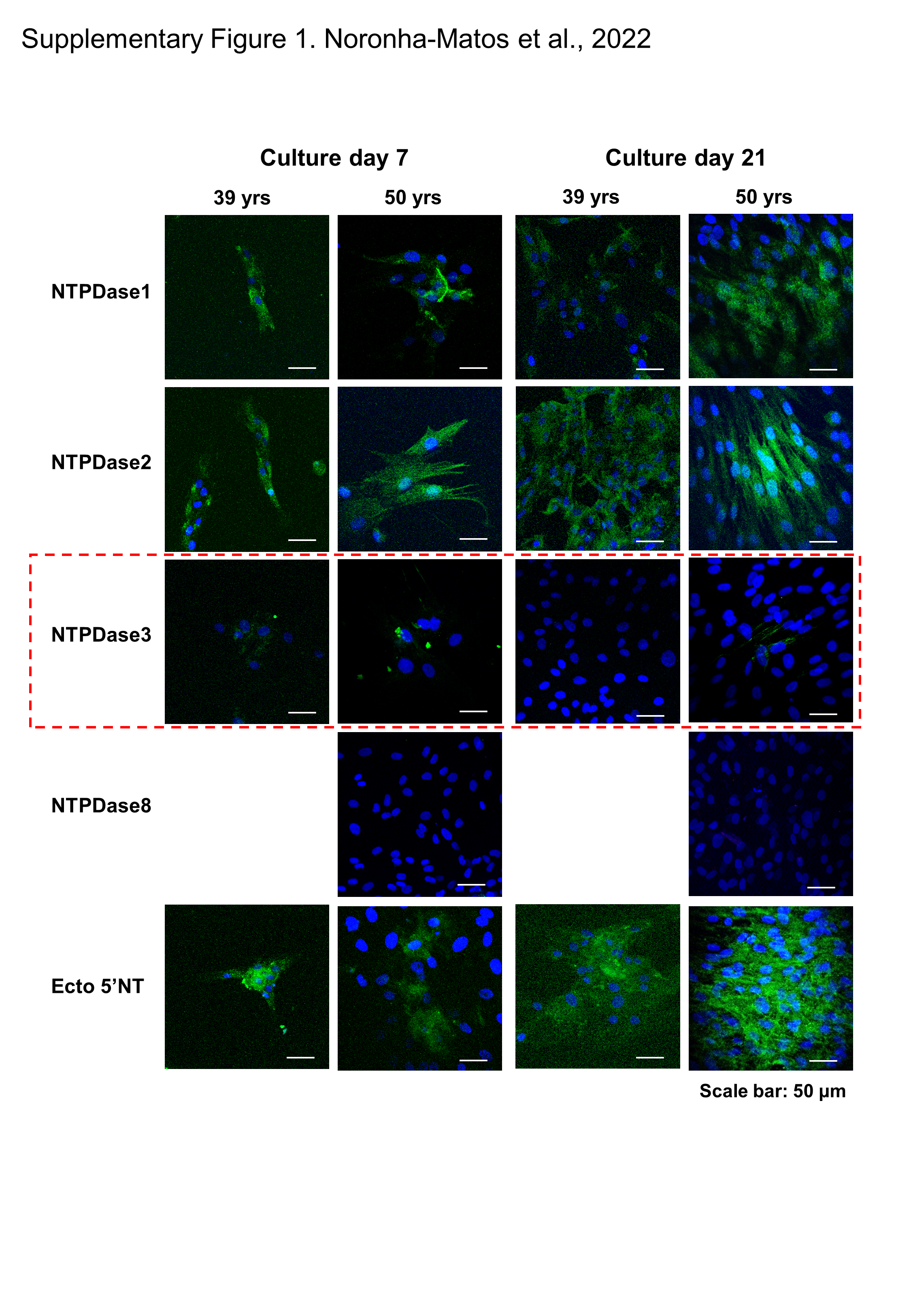

Supplement: Supplementary file 3 — Additional file 3. Fig. S1 Immunocytochemical detection of NTPDase1, -2, -3, -8 and ecto-5’-nucleotidase (Ecto 5’-NT) in cultured BM-MSCs (first subculture) from young (Y) and older (O) males, which were allowed to grow for 7 and 21 days in an osteogenic-inducing medium. Blue dots represent nuclei stained with DAPI. These experiments were performed in parallel to those using women's samples shown in Figure 1 while keeping unaltered the settings of the confocal microscope throughout the experimental procedure (see Materials and Methods). The scale bar is 50 µm [file 13287_2023_3315_MOESM3_ESM.tif]

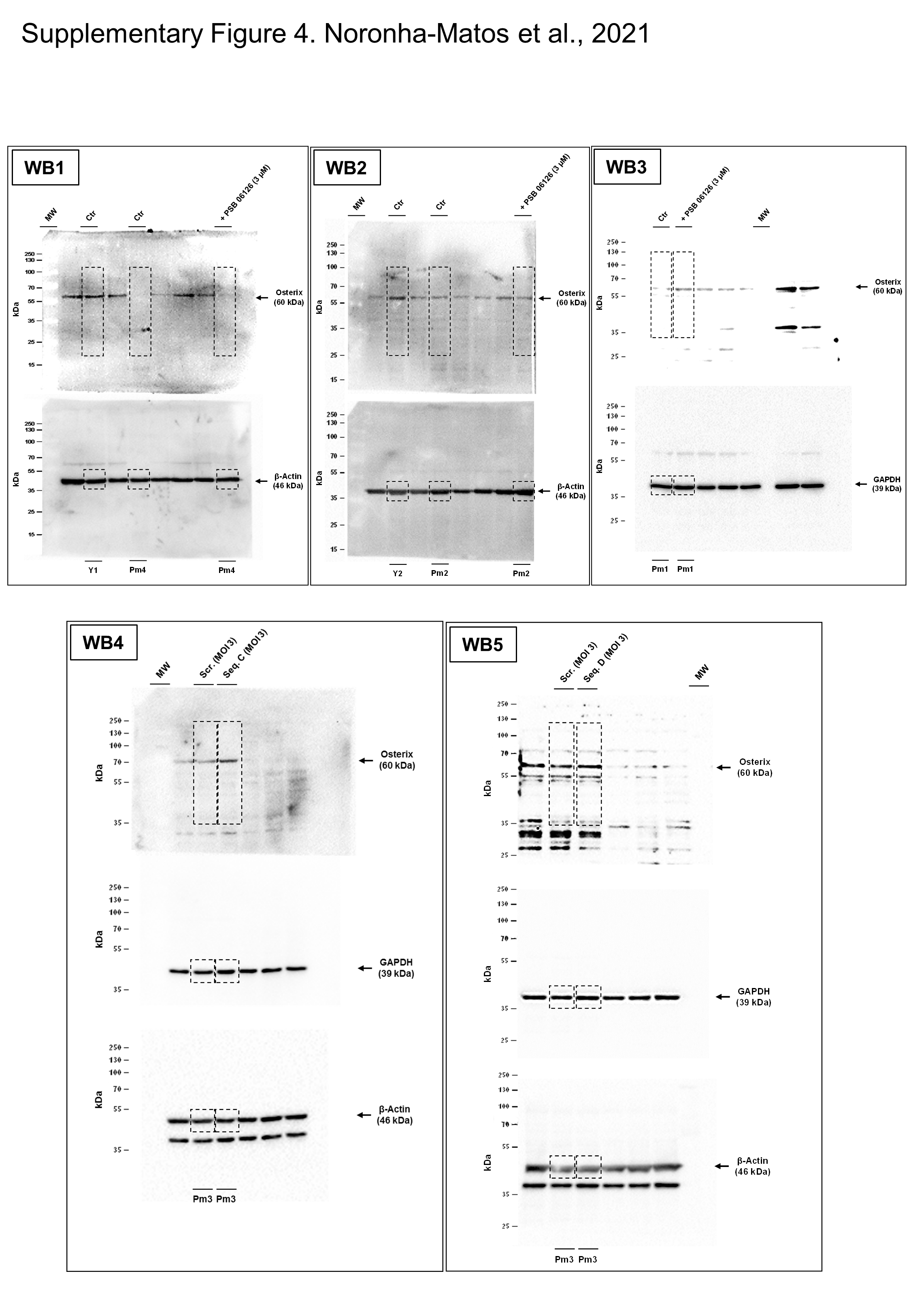

Supplement: Supplementary file 4 — Additional file 4. Fig. S4 Uncropped full-length Western-blot gels depicted in Figure 7A (WB1-5). Shown are typical immunoblots stained for Osterix (~60 kDa) transcription factor in BM-MSCs from two young (Y1 and 2) and three Pm (Pm1, 2 and 3) women cultured in an osteogenic-inducing medium (i) in the absence or the presence of the NTPDase3 inhibitor, PSB 06126 (3 µM), or (ii) after NTPDase3 gene silencing with lenti-shRNAs encoding for TL313202VD (Seq D; MOI 3) and TL313202VC (Seq C; MOI 3) inhibitory vs. control scramble sequences; either β-Actin (46 kDa) or GAPDH (39 kDa) protein amounts were used as house-keeping gene product standards for normalization purposes. Dashed boxes indicate where blots were cropped for comparison purposes. [file 13287_2023_3315_MOESM4_ESM.tif]
